# The Targeted Inhibition of Histone Lysine Demethylases as a Novel Promising Anti-Cancer Therapeutic Strategy—An Update on Recent Evidence

**DOI:** 10.3390/cancers17172798

**Published:** 2025-08-27

**Authors:** Jarosław Paluszczak, Robert Kleszcz

**Affiliations:** Department of Pharmaceutical Biochemistry, Poznan University of Medical Sciences, 60-806 Poznań, Poland; kleszcz@ump.edu.pl

**Keywords:** epigenetics, cancer, histone lysine demethylases, KDM, LSD1, KDM4, KDM5, KDM6, anti-cancer therapy

## Abstract

A growing body of evidence confirms that non-mutational epigenetic reprogramming constitutes an important hallmark of cancer, contributing to the heterogeneity and phenotypic plasticity observed in cancers. Among the many epigenetic modulators, histone lysine demethylases (KDMs) have emerged as promising targets for pharmacological inhibition in cancer treatment. KDMs were found to be frequently overexpressed and/or hyperactivated in cancer cells, and their inhibition was shown to result in the inhibition of cancer cell growth both in vitro and in vivo. The inhibition of Lysine-specific histone demethylase 1A (LSD1), KDM3, KDM4, KDM5, and KDM6 may affect cell survival, proliferation, motility, and apoptosis induction. Importantly, KDM inhibitors can be used as modulators of anti-cancer immune response and sensitivity to radiation and chemotherapy. This narrative review aims to present the most recent evidence documenting the anti-cancer potential of KDM inhibitors.

## Simple Summary

The dysregulation of epigenetic mechanisms is an important feature of cancer cells. Histone lysine demethylases affect the expression of many genes responsible for the regulation of cell survival, proliferation, motility, metabolism, or cell death. The inhibition of these proteins can significantly block the proliferation of cancer cells and the growth of tumors. This is also associated with the effects on the tumor microenvironment and immune surveillance. The aim of this narrative review is to present recent evidence of the therapeutic utility of the inhibition of histone lysine demethylases for cancer treatment.

## 1. Introduction

The field of cancer epigenetics is quickly expanding, and a growing body of evidence supports the assumption that non-mutational epigenetic reprogramming constitutes an important hallmark of cancer, which can contribute to the acquisition of key alterations necessary during tumor development and malignant progression [1]. Many epigenetic writers, readers, erasers, and remodelers are engaged in the complex modulation of gene expression, and they collectively contribute to the heterogeneity and phenotypic plasticity observed in cancers [2]. Indeed, cancer cells are characterized by altered epigenetic states: aberrant profiles of DNA methylation, and abnormal patterns of post-translational modifications of histones. Besides reversible acetylation, phosphorylation, ubiquitylation, lactylation, etc., histones can undergo reversible methylation on lysine (K) or arginine (R) residues by histone methyltransferases (e.g., EZH2 catalyzing H3K27 methylation), which can have diverse biological effects, depending on the localization of the residue and the level of its methylation, since lysine residues can be mono-(me1), di-(me2), or tri-methylated (me3), while arginine residues can undergo mono-methylation, or symmetrical or asymmetrical dimethylation. In general, transcriptional activation is mediated by the methylation of histone H3 on lysine K4 (H3K4me2/3) within gene promoter regions and around transcription start sites, and by the methylation of histone H3 on lysine K36 within coding regions. Transcriptional repression is mediated by the methylation of lysine 9 or lysine 27 on histone H3 (H3K9me2/3, H3K27me2/3) in gene promoters and around transcription start sites [3,4]. Methylation marks on histones can be removed by a group of histone lysine demethylases (KDMs), which are divided into two families, based on the co-factors required for catalysis. There are two FAD-dependent demethylases (KDM1A/B, or more commonly—LSD1/2). Histone demethylases from the second group (KDM2-7) contain a conserved Jumonji C (JmjC) catalytic domain, and these dioxygenases require Fe^2+^, 2-ketoglutarate, and oxygen for their activity [5,6]. Each KDM shows preference for a particular site/residue and methylation level, allowing for a coordinated regulation of histone methylation profiles. This is achieved in complex interaction with a wide set of other modulatory proteins [2].

Evidence is accumulating regarding the significance of KDMs for the pathogenesis of cancer, and the therapeutic efficacy of the inhibition of KDMs [4,5,7,8]. Thus, the aim of the present paper was to review the most recent research related to the anti-cancer potential of KDM inhibition. The PubMed database search using “histone lysine demethylase inhibition cancer” as keywords retrieved around 1400 records, and we have identified relevant papers published between 2022 and 2025. This narrative review discusses the therapeutic potential of KDM inhibitors (KDMi), both as individually applied treatments, and used in combinations with other therapeutic approaches. While no KDMi has been approved for the treatment of cancer patients so far, clinical trials evaluating the effects of several LSD1 or KDM4 inhibitors are under way [8], and clinical oncology may be approaching the inclusion of KDMi into the anti-cancer therapeutic armamentarium.

## 2. The Inhibition of Histone Lysine Demethylases Has Anti-Cancer Effects

Most histone lysine demethylases have become important molecular targets which can be pharmacologically inhibited to exert anti-cancer effects in many tumor types [3]. The inhibition of LSD1 attracted the most attention; however, inhibitors targeting each KDM have been developed in recent years. Most of the small molecule inhibitors target the enzymatic activity of KDMs, which may not fully recapitulate the effects of KDM knockdown, since the biological function of KDMs is partly determined by protein–protein interactions irrespective of histone demethylation [9]. Thus, the emergence of the group of PROTAC (proteolysis-targeting chimera) degraders, which selectively reduce the cellular level of KDM proteins by stimulating their ubiquitin-dependent proteasomal degradation, may add new information in the field [6,10,11,12]. Although rarely reported, the observed action of KDMs may sometimes be related to activity towards non-histone substrates, e.g., LSD1 was shown to demethylate the LC3B autophagy-related protein, thus promoting its degradation [13].

**Figure 1:**
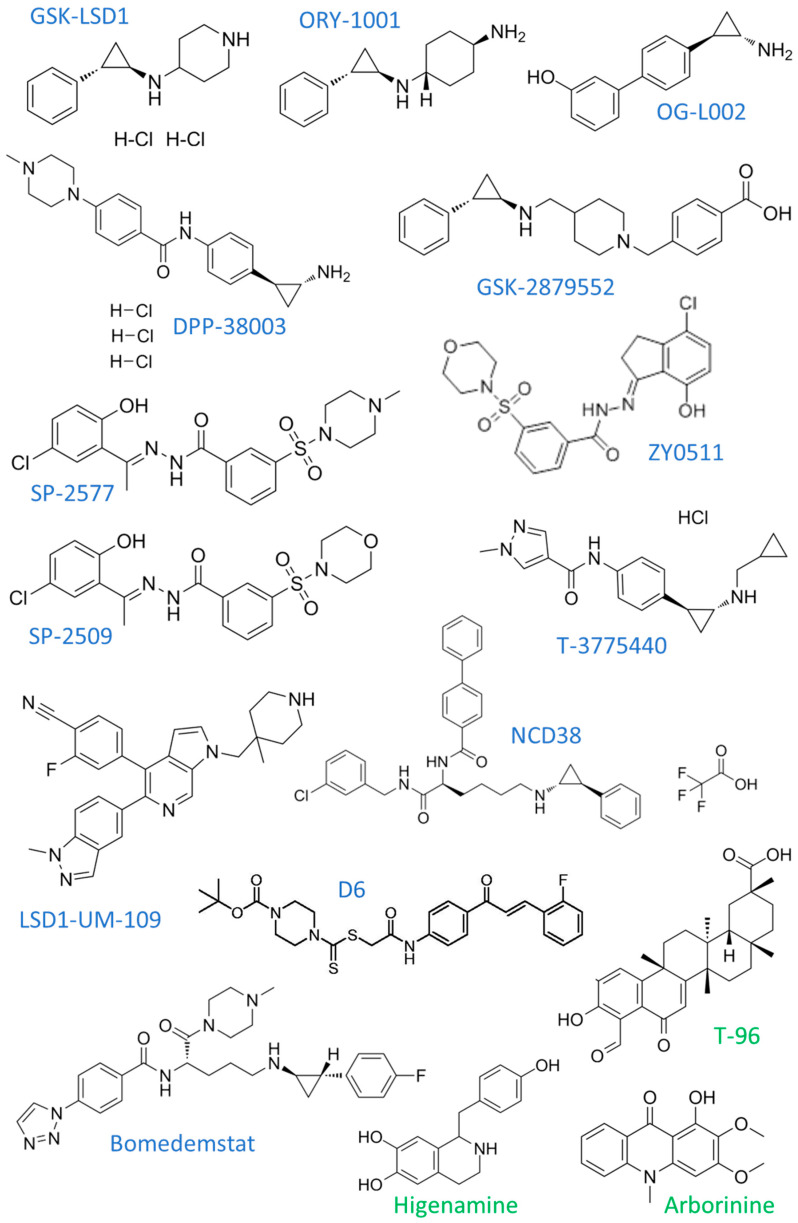
The chemical structures of synthetic (names in blue) and natural (names in green) LSD1 inhibitors. Source: medchemexpress.com.

**Figure 2:**
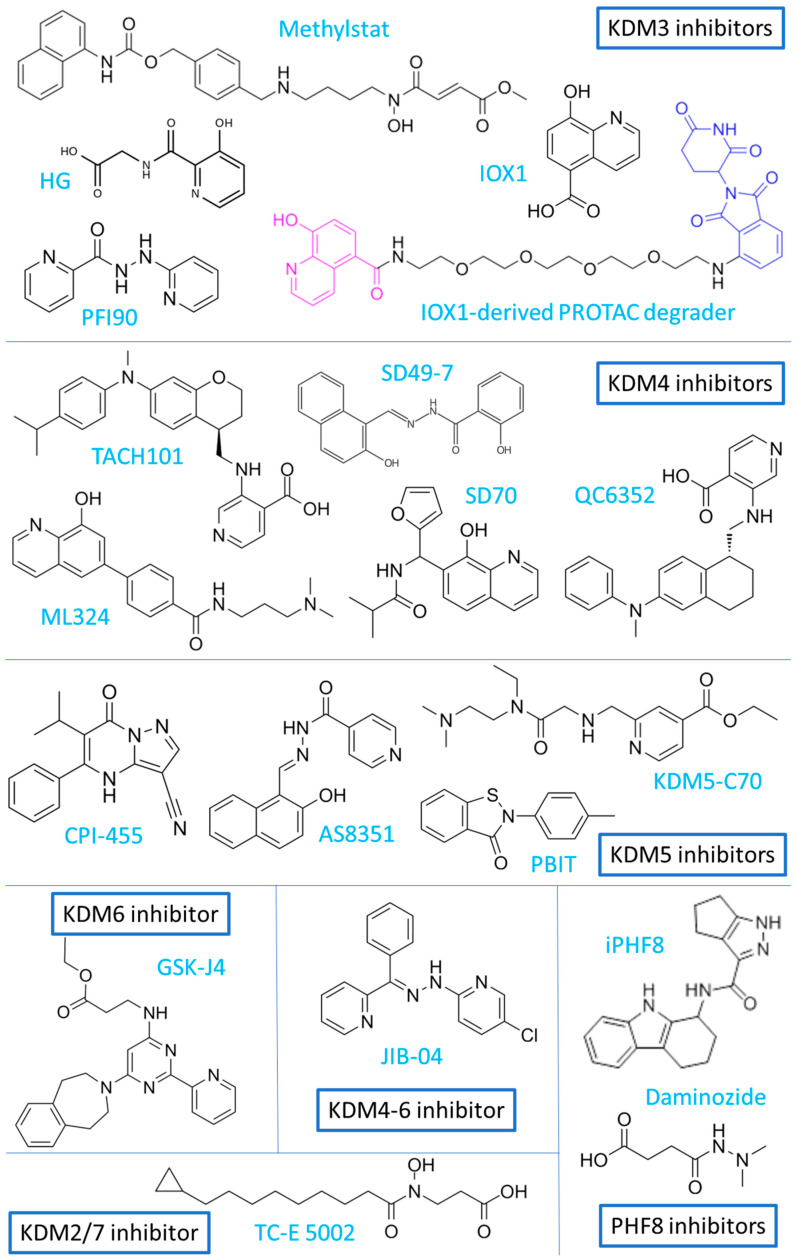
The chemical structures of JmjC-type KDM inhibitors. It must be remembered that KDM3 inhibitors shown in the figure are not selective towards KDM3. Source: medchemexpress.com.

Not only synthetic chemicals, but also natural compounds, can significantly modulate the activity of various epigenetic proteins, including histone lysine demethylases. Relevant information regarding this topic can be found in a recent review, and Table 1 presents latest evidence which was not included in the paper [14]. In the following sections we focus on the characterization of the biological effects of KDM knockdown, and the activity of synthetic KDM inhibitors (KDMi). Table 2 presents a summary of the recent findings on the pharmacological inhibition of KDMs in cancer models. The chemical structures of most of the inhibitors mentioned in the text are presented in Figure 1 and Figure 2.

### 2.1. KDM1A/B (LSD1/2)

LSD1 is the first discovered histone lysine demethylase [4]. KDM1 proteins catalyze the demethylation of H3K4me1/2 and H3K9me1/2, and act as transcriptional repressors [7,8]. Detailed studies of the role of LSD1 in cancers resulted in LSD1i entering clinical trials [8]. Importantly, alterations in LSD1 expression and activity were observed during chemically induced carcinogenesis and suggest a partly causal role. Bladder cancer induced by N-Butyl-N-(4-hydroxybutyl) nitrosamine (OH-BBN) was associated with elevated expression of LSD1 and decreased level of H3K4me, while normal bladder epithelium was characterized by the absence of LSD1. In parallel, the immunohistochemical analysis pointed to increased LSD1 protein level in human urothelial cancer tissue samples [23]. In a mouse model of oral squamous cell carcinoma (OSCC) induced by exposure to carcinogenic 4-nitroquinoline 1-oxide (4NQO), the conditional deletion of *Lsd1* attenuated tongue tumor development. On the molecular level, this was associated with reduced Egfr and Jnk signaling, and decreased Il-6 expression. Moreover, tumors were characterized by reduced proportion of M2 macrophages, and increased *Pd-l1* expression, pointing to important changes in the tumor microenvironment (TME) and immune response. Pharmacological inhibition of Lsd1 with SP2509 also reduced tumor burden in this model. The activity of SP2509 was further enhanced by combining it with verteporfin, a YAP inhibitor, which points to important interconnections with the Hippo pathway [33]. In a subsequent study it was found that *Kdm1a* knockout significantly reduced the risk of OSCC development upon 4NQO treatment in experimental mice, and the application of LSD1 inhibitor SP2509 during dysplasia prevented progression towards neoplastic lesions. It was hypothesized that the effects were mediated by the modulation of the activity of STAT3 signaling pathway [34].

The silencing of LSD1 by shRNA in FaDu hypopharyngeal cancer cells decreased cell proliferation, migration, invasion, and epithelial-to-mesenchymal transition (EMT), which was related to induced autophagy and pyroptosis [35]. On the other hand, targeting LSD1 in acute lymphoblastic leukemia cells had a dual role in cell migration and invasion control: promoting invasiveness in 3D environments but reducing extravasation and chemotaxis in vivo [36]. The siRNA-mediated depletion or pharmacological inhibition (GSK2879552) of LSD1 reduced tumor growth in an orthotopic model of prostate cancer, and the effect was potentiated by concurrent castration. Moreover, GSK2879552 reduced the migration and invasion of prostate cancer cells in vitro, leading to the downregulation of MMP13 expression. In addition, it reduced the metastatic potential of prostate cancer cells in vivo [37]. LSD1 was also found to play important roles in the regulation of EMT in non-small cell lung cancer (NSCLC) cells. TdT-interacting factor 1 (TdIF1) was shown to regulate the binding of LSD1 to *CDH1* promoter region, and TdIF1 knockdown reduced LSD1 occupancy and increased the level of H3K4me2 around *CDH1* promoter, leading to increased expression of E-cadherin. The combined treatment of NSCLC cells with SP2509 LSD1 inhibitor and siRNA against TdIF1 synergistically led to reverting the cadherin switch, causing the upregulation of the expression of E-cadherin and downregulation of N-cadherin, which were associated with diminished cell invasion [38].

The cellular level of LSD1 protein can be regulated by its ubiquitination and proteasomal degradation. It was shown that FBXO24, an E3 ligase, catalyzed LSD1 ubiquitination in breast cancer cells. The depletion of LSD1 by FBXO24 led to the reduction in stemness potential; it decreased the proportion of CD44-positive stem-like cells and reduced the expression of stemness-related genes *SOX2* and *OCT4*. In addition, FBXO24-mediated depletion of LSD1 level reduced breast cancer cell migration and invasion, which was associated with the increased expression of E-cadherin and reduced expression of Vimentin. Furthermore, the overexpression of FBXO24 attenuated breast xenograft tumor growth in vivo [39]. On the other hand, LSD1 deubiquitination catalyzed by USP1 stabilized LSD1 protein in triple-negative breast cancer (TNBC) cells, and USP1 depletion led to decreased cellular LSD1 level, and led to pharmacological effects similar to LSD1 depletion, such as negatively affected cell proliferation, migration, invasion, sphere formation, and induced apoptosis [40]. Another study also pointed to the association between LSD1 and stemness potential: LSD1 knockdown was shown to decrease the expression of breast cancer stem cell signature genes (*SOX2, SOX9, OCT4*), which was mediated by the interaction between LSD1 and RBBP7. Interestingly, breast cancer cell lines showing high level of RBBP7 expression were more sensitive to LSD1 inhibitor ORY-1001 (iadademstat), which effectively reduced the expression of stemness genes and *CCND1*. This was confirmed in vivo: ORY-1001 reduced the growth of RBBP7^high^ (but not RBBP7^low^) patient-derived organoids. Moreover, ORY-1001 reduced the metastatic potential of RBBP7^high^ tumors [41]. LSD1 was also important for the regulation of stemness potential via modulation of the canonical Wnt pathway in thyroid cancer cells [42].

A recent interesting study showed the differential response of glioblastoma cell lines to the pharmacological inhibition of LSD1. It was shown that quiescent cells characterized by lower glycolytic activity were not sensitive to LSD1i, and it was revealed that PGAP1 mediated resistance to LSD1i. Indeed, PGAP1 knockdown sensitized resistant cells to LSD1i and resulted in additive cytotoxic effects of the combination of LSD1i and temozolomide [43]. The development of resistance to the pharmacological inhibition of LSD1 was also observed in a xenograft mouse model of glioblastoma. While GSK-LSD1 treatment led to a delayed but significant reduction in tumor burden in experimental animals, the anti-cancer effects gradually disappeared over time. The upregulated expression of *FAM213A*, *FTH1*, *HKDC1*, and *RAB39B* was suggested as a marker of resistance to LSD1 inhibition [44].

The susceptibility to KDM inhibition may significantly differ between cancer types and subtypes, and this topic requires further elucidation. For example, D6 chalcone derivative—an LSD1 inhibitor—potently affected the viability of leukemic cells but was much less effective against cervical or ovarian cancer cells [45]. On the other hand, another study reported that the knockdown of LSD1 in ovarian cancer cells led to anti-cancer effects both in vitro and in vivo, attenuating cell motility, causing apoptosis induction, and reducing tumor weight and volume in xenograft mice [46]. In line with this observation, the expression of LSD1 was shown to correlate with unfavorable prognosis and shorter overall survival in ovarian cancer patients [13]. Overall, LSD1 promotes the survival, proliferation, stemness, and motility of neoplastic cells of various origin, which makes it a good pharmacological target in many cancer types.

**Table 2:** Synthetic inhibitors of histone lysine demethylases and their biological effects.

Name of Compound	Target KDM	Biological Effects	Reference
4,5-Dimethoxycanthin-6-on	LSD1	Reduced glioblastoma cell viability, migration, and colony formation; decreased activity of AKT/mTOR and MAPK signaling pathways; induction of apoptosis and pyroptosis; reduced tumor weight and volume in mouse U251 cells xenograft	[47]
B35 (indole derivative)	LSD1	Inhibited lung cancer cell proliferation and colony formation; induced apoptosis; reduced tumor volume and percentage of Ki-67 positive cells in mouse A549 cells xenograft	[48]
Compound 5a (cyanopyrimidine derivative)	LSD1	IC50 values < 10 µM in various cell lines, e.g., leukemia, non-small cell lung cancer, and colorectal cancer cells; anti-inflammatory potential based on TNF-α reduction in RAW 264.7 murine monocytes	[49]
Compound 9j (tranylcypromine-based triazolopyrimidine analog)	LSD1	Viability reduction in gastric cancer, lung cancer, and prostate cancer cell lines; inhibition of epithelial-to-mesenchymal transition; S-phase cell cycle arrest; apoptosis induction; reduced tumor volume and weight in mouse H1650 lung cancer cells xenograft	[50]
Compound 10e (sulfonyl benzoyl hydrazide derivative)	LSD1	Viability reduction in lung cancer, colorectal cancer, ovarian cancer, and breast cancer cell lines; induction of apoptosis and S-phase cell cycle arrest in HCT116 cell line; decreased tumor volume and weight in mouse HCT116 cells xenograft	[51]
DDP-38003	LSD1	Reduced glycolytic capacity, ER stress induction, and reduced tumor growth in the zebrafish larvae model of glioblastoma tumor-initiating cells; resistance to LSD1i depended on PGAP1 expression	[43]
D6 (chalcone derivative)	LSD1	D6 derivative potently inhibited LSD1 and led to accumulation of H3K9me1/2 in leukemia cancer cells; D6 reduced cell proliferation in vitro and suppressed xenograft tumor growth in vivo	[45]
GSK-LSD1	LSD1	Reduced tumor growth in a xenograft model of thyroid cancer	[42]
LSD1-UM-109	LSD1	LSD1-UM-109 (compound 46)—a pyrrolo [2,3-c]pyridine derivative—showed strong inhibition of acute myeloid leukemia and squamous cell lung cancer cell proliferation	[52]
NCD38	LSD1	Reduced triple-negative breast cancer cell viability, invasion, and mammosphere formation; induced apoptosis; downregulated IL-6/JAK/STAT3 and EMT pathways; reduced tumor volume and weight in mouse breast cancer patient-derived cells xenograft	[53]
ORY-1001	LSD1	Reduced proliferation and induced apoptosis in hepatocellular cancer cells	[15]
S2172	LSD1	Reduced proliferation of glioblastoma cells and impaired growth of xenograft tumors; significantly increased H3K4me1/2/3 and H3K9me2 level; reduced expression of cancer stem cell markers; S2172 penetrates the blood-brain-barrier	[54]
SP2509	LSD1	Reduced tongue tumor development induced by 4NQO in a mouse model	[33]
PFI-90	LSD1, KDM3B	Suppressed xenograft tumor growth of *PAX3-FOXO1* fusion-positive rhabdomyosarcoma, delayed tumor progression	[55]
IOX1	KDM3	Reduced proliferation of CRC cells, reduced stemness potential, and inhibited xenograft tumor growth; inhibited Wnt target gene expression	[56]
	KDM2A/3A	Reduced proliferation and invasion, and increased apoptosis in bladder cancer cells; decreased tumor volume in xenograft mice	[57]
IOX1-based PROTAC * #4	KDM3	Decreased Wnt signaling pathway activity and Wnt target genes expression in colorectal cancer cell lines, reduced the growth of CRC stem cells-derived tumors in vivo	[10]
ML324	KDM4	Reduced bladder cancer cell proliferation, and induced apoptosis; decreased tumor growth in mouse bladder cancer cells xenograft	[58]
SD49-7	KDM4	Reduced leukemia cell viability in vitro and leukemia development in the patient-derived cells xenograft mouse model; increased apoptosis by suppressing MDM2 expression via change in H3K9me3 levels on the *MDM2* promoter region	[59]
TACH101	KDM4A-D	Viability reduction in leukemia, breast cancer, esophageal cancer, multiple myeloma, colorectal cancer, and breast cancer cells; reduced tumor volume in various mouse xenografts	[60]
QC6352	KDM4	Significantly delayed MYCN-amplified neuroblastoma growth in a patient-derived xenograft model	[61]
Compound 33a (4,6-diarylquinoxaline derivative)	KDM4D	Reduced liver cancer cell proliferation, colony formation, and migration	[62]
AS-8351	KDM5B	Inhibited Ewing sarcoma cells in vitro and in vivo via upregulation of FBXW7 and enhanced degradation of cyclin E1	[63]
Compound 11ad (1H-pyrazole- [3,4-b] pyridine derivative)	KDM5B	Reduced prostate cancer cell viability, colony formation, and migration with concomitant induction of apoptosis and G2/M cell cycle arrest via attenuation of PI3K/AKT signaling pathway	[64]
KDM5-C70	KDM5B	Reduced expression of PPARγ coactivator-1α (PGC1α) and prostate-specific antigen, followed by decreased cell proliferation of prostate cancer cell lines	[65]
GSK-J4	KDM6A/B	Different effects in mouse prostate cancer cells xenografts: decreased tumor growth in androgen-dependent cancer and increased tumor volume in androgen-independent cancer	[66]
	KDM6A/B	Reduced retinoblastoma cell viability, proliferation, colony formation, and PI3K/AKT/NF-κB signaling pathway-related proteins; decreased tumor volume and Ki-67 expression in mouse xenograft	[67]
	KDM6A/B	Reduced cervical cancer cell viability, colony formation, migration, and invasion; decreased tumor growth in mouse xenografts	[68]
	KDM6B	Reduced adhesion of mantle lymphoma cells to stromal cells by downregulating the NF-κB pathway	[69]
	KDM6B	Reduced prostate cancer cell viability; cytotoxicity; reduced cell migration and invasion; inhibition of canonical TGFβ pathway	[70]
	KDM6B	In vitro reduced invasion of osteosarcoma cells; in vivo reduced lung metastasis (mouse 143B cells xenograft)	[71]
JIB-04	KDM4–6	In vitro reduced breast cancer cell migration and colony formation with concomitant lower expression of EMT—supporting proteins N-cadherin, Vimentin, and Snail; in vivo decreased tumor volume in mouse MDA-MB-231 cells xenograft	[72]
	KDM4, KDM6	Reduced viability, migration, invasion, and stemness in hepatocellular cancer cells, reduced E2F-dependent transcription of cell cycle regulatory genes by decreasing AKT expression via KDM6B	[73]
Compound 4	KDM7A	Reduced breast cancer cell viability, including taxol-resistant cells; G1/G0 cell cycle arrest; decreased expression of stemness markers	[74]
TC-E 5002 (KDM2/7-IN-1)	KDM7A	Overcoming of cisplatin-resistance in mouse T24 cells bladder cancer xenograft	[75]
Daminozide	PHF8	Promoted differentiation of chronic myeloid leukemia cells, reduced cell proliferation, decreased the expression of *BCR-ABL1*	[76]
iPHF8	PHF8	Reduced HCT-116 cell proliferation in vitro; reduced xenograft tumor growth	[77]

* PROTAC—proteolysis-targeting chimera.

### 2.2. KDM2A/B (JHDM1A/B)

KDM2 proteins catalyze the demethylation of H3K36me1/2 and H3K4me3 (KDM2B), and act as transcriptional repressors [3,8]. Few studies focused on the exploration of the significance of KDM2 proteins in cancer cells, and no selective KDM2 inhibitors have been developed so far. However, evidence supports the pro-tumorigenic role of KDM2A/B. Targeting KDM2B by siRNA negatively affected lung squamous cell carcinoma cells in vitro and in vivo via the inhibition of PI3K/AKT/mTOR signaling and glycolysis [78]. Moreover, shRNA against KDM2B reduced proliferation, migration, and invasion, and induced apoptosis of NSCLC cells by inhibiting the EZH2/PKMYT1/Wnt/β-catenin axis. In addition, it reduced tumor growth in experimental xenograft mice, leading to reduced β-catenin and Ki-67 expression [79].

### 2.3. KDM3 (JMJD1)

KDM3 proteins catalyze the demethylation of H3K9me1/2, and act as transcriptional activators [3]. KDM3A activity supported the malignancy of TNBC [80] and OSCC cells [81]. On the other hand, KDM3B was found to be crucial in maintaining genome stability in melanoma cells, and *KDM3B* knockout increased DNA damage, and enhanced cell growth and migration [82]. In contrast, the knockdown of KDM3B in *PAX3-FOXO1* fusion-positive rhabdomyosarcoma cells reduced the expression of PAX3-FOXO1 target genes by increasing H3K9 methylation within gene promoter regions. Moreover, the concurrent knockdown of LSD1 and KDM3B led to enhanced apoptosis, and phenocopied the effects of action of PFI-90 small molecule KDM inhibitor [55]. Another study showed that KDM3A/B were recruited to Wnt target gene promoters in colorectal carcinoma (CRC) cells, and they contributed to transcriptional gene activation by demethylating H3K9me2/3. Since colorectal carcinogenesis is frequently related to canonical Wnt/β-catenin pathway activation, the downregulation of KDM3 activity could exert anti-cancer effects in CRC. Indeed, IOX1 inhibitor led to enrichment of H3K9me2/3 in Wnt target gene promoters, and decreased cell proliferation, migration, and stemness potential. Interestingly, stronger inhibition of expression of Wnt target genes was observed in stem-like ALDH^high^ subpopulation of cells, which were characterized by elevated level of KDM3A/B proteins. Accordingly, IOX1 reduced tumor size in xenograft tumors derived from ALDH^high^ cells [56]. IOX1 reduced the viability, proliferation, migration, and induced apoptosis in colorectal cancer cells. It also reduced xenograft tumor growth in vivo [83].

### 2.4. KDM4 (JMJD2)

KDM4 proteins catalyze the demethylation of H3K9me2/3 and H3K36me2/3, and act as transcriptional repressors [3,8]. KDM4s have been implicated in the tumorigenesis in various tissues, and their inhibition was shown to affect cell viability, proliferation, migration, invasion, and tumor growth in vivo. The knockout of *KDM4A* or *4B* or *4C* reduced neuroblastoma cell proliferation in vitro and tumor growth in vivo [61]. In a similar manner, the induction of KDM4A-C degradation with the use of RDN8011, a TACH101 derivative acting as a selective PROTAC degrader, showed effective reduction in cell proliferation and induction of apoptosis in esophageal squamous cell carcinoma (ESCC) cells [12].

The deletion of KDM4A reduced esophageal xenograft tumor growth and metastatic capacity [84]. In OSCC cells, KDM4A is recruited by LEF1 transcription factor (a downstream effector of the canonical Wnt signaling pathway) and binds the large tumor suppressor kinase 2 promoter, inhibiting its expression and, as a consequence, promoting OSCC cell proliferation and avoidance of apoptosis. Moreover, shRNA-mediated KDM4A knockdown led to a significant reduction in xenograft tumor growth in vivo [85]. In breast cancer, KDM4A was shown to promote xenograft tumor growth and stimulate lung metastases in experimental mice. These effects on tumor progression were mediated by H3K9me3 demethylation-related activation of Notch signaling, and breast cancer cells characterized by high KDM4A expression were much more sensitive to Notch inhibition [86]. Furthermore, KDM4A was implicated in the regulation of androgen receptor (AR) expression in prostate cancer cells by the epigenetic modulation of AR enhancer. KDM4A knockdown downregulated AR protein level and activity, and attenuated prostate tumor growth in a patient-derived xenograft model [87]. Also the expression of KDM4C was higher in metastatic prostate cancer patients, and KDM4C knockdown or pharmacological inhibition using SD70 significantly reduced cell migration and invasion, which was associated with the increased level of E-cadherin [88].

High expression of KDM4B was also associated with poor prognosis in glioblastoma patients. Accordingly, shRNA-mediated KDM4B knockdown reduced xenograft tumor growth. Mechanistically, these effects were shown to be mediated by the KDM4B-induced stabilization of MYC transcription factor, and KDM4B knockdown led to reduced MYC level, and subsequent downregulation of MYC-related expression of cell cycle-promoting and EMT-related proteins [89]. The overexpression of KDM4B was associated with increased tumor size and enhanced risk of liver metastasis in a mouse xenograft model of renal clear cell carcinoma (RCCC). The knockdown of KDM4B reduced RCCC cell proliferation, migration, and invasion in vitro [90]. KDM4B was also upregulated in primary and metastatic melanomas. The inhibition of KDM4B with NCGC00244536 led to cell cycle arrest and apoptosis induction in melanoma cells, irrespective of p53 status [91].

The knockdown of KDM4C led to reduced stemness features, and reduced cell migration and invasion in TNBC cells. Moreover, it reduced cell metastasis in vivo. These effects were related to the elevation of promoter H3K9me3 level and transcriptional downregulation of the expression of KLF14 transcription factor, which caused a reduction in cellular cholesterol content [27]. Interestingly, it was shown that the depletion of KDM4B or 4D, but not 4A or 4C, reduced the stemness potential of hepatocellular carcinoma cells [73]. On the other hand, shRNA-mediated knockdown of KDM4C reduced hepatocellular xenograft tumor growth, and KDM4C silencing reduced cell migration and expression of proteins regulating cell motility—ZEB1 and Snail, in vitro. These effects were associated with the KDM4C-dependent modulation of H3K36 methylation level in the promoter region of *CXCL2* [92]. KDM4C silencing in HNSCC cells reduced cell migration in vitro, attenuated invasion and metastasis in zebrafish, and impaired xenograft tumor growth in mice. Such action was related to the reduction in ferrochelatase (FECH) expression, and the effects could be reversed by FECH overexpression [32].

The inhibition of KDM4D was suggested as a potential novel target for the treatment of imatinib-resistant gastrointestinal stromal tumors. The reduction in KDM4D demethylase activity by upregulated miR-409-5p inhibited HIF1β and VEGF-A expression, and inhibited angiogenesis. In addition, this treatment synergized with imatinib in reducing tumor growth in vivo [93].

Overall, the pro-tumorigenic role of KDM4s is well-documented in many cancer types, and TACH101—a KDM4 inhibitor—is the first inhibitor of JmjC demethylases that entered clinical trials [8].

### 2.5. KDM5 (JARID1)

KDM5 proteins catalyze the demethylation of H3K4me2/3, and act as transcriptional repressors [3,8]. Changes in the activity of KDM5 proteins may be causally related to carcinogenesis. A recent study showed that skin squamous cell carcinomas induced by inorganic arsenic were associated with the increased expression of several epigenetic proteins (histone demethylases: KDM5B, and histone methyltransferases: EZH2, MLL3) and exhibited reduced levels of H3K4me3 and elevated levels of H3K27me3. Importantly, the development of tumors in this model was prevented by black tea extract. Mechanistically, the chemopreventive effect exerted by teaflavin compounds could depend on the modulation of the activity of KDM5B, which was suggested by in silico analyses [94].

KDM5A promoted prostate adenocarcinoma progression via suppression of miRNA-300-3p, followed by restored coatomer protein complex subunit β2 expression and activation of PI3K/AKT signaling. Knockdown of KDM5A reduced cell proliferation, migration, and invasion, and induced apoptosis [95]. Moreover, this lysine demethylase enhanced the expression of mesenchymal Vimentin, thus promoting EMT [96]. KDM5A was upregulated in osteosarcoma patients and was associated with poor prognosis—shorter survival and more frequent recurrence and metastasis. The knockdown of KDM5A reduced cell proliferation and migration, induced apoptosis in vitro, and also reduced xenograft tumor growth in vivo. These effects were associated with the downregulation of c-MYC and Wnt/β-catenin pathway activity. In addition, increased HOXA5 expression due to accumulation of H3K4me2/3 was observed [97]. Another study also pointed to the association between KDM5B and Wnt/β-catenin signaling in pancreatic cancer. The patients showed elevated KDM5A/B expression which predicted poor survival [98].

KDM5B promoted metastasis and invasion of HNSCC cells via the Wnt/β-catenin pathway [99]. Interestingly, osteosarcomas showing the presence of *KMT2D* tumor suppressor loss-of-function mutations were sensitive to KDM5B inhibition. KDM5B knockdown or small molecule inhibitors showed much stronger effects towards *KMT2D*-depleted cells than wild type cells in vitro, leading to reduced cell proliferation, and invasion. In parallel, the pharmacological inhibition of KDM5B using AS8351 was much more potent in reducing *KMT2D*-depleted xenograft tumor growth [100]. In breast cancer cells the response to KDM5 inhibitors was mediated by the level of expression of ZBTB7A transcription factor, and ZBTB7A depletion sensitized basal breast cancer cells to KDM5i [101].

The RNA and protein level of KDM5B was elevated in localized and advanced prostate carcinomas, and it was shown that KDM5B together with LSD1 was implicated in the regulation of expression of AR target genes. The combined inhibition of KDM5B and LSD1 with the use of CPI-455 and namoline, respectively, led to reduced expression of AR-responsive genes and reduced cell proliferation and invasion in both castration-sensitive and castration-resistant prostate cancer cells [102]. KDM5B, by decreasing H3K4me3 level, reduced the transcription of *ETS homologous factor* (*EHT*), and further reduced the transcriptional activation of *Filamin-B*. Both EHT and FLNB are molecules related to immune evasion in RCCC. Therefore, high activity of KDM5B promoted RCCC. Importantly, KDM5B knockdown reduced tumor growth in vivo [103].

It was found that KDM5C expression was elevated in colorectal cancer patients, and high expression correlated with shorter relapse-free survival and with lymph node involvement. Moreover, KDM5C was functionally associated with stimulation of cell proliferation, migration, and invasion in CRC cells. The knockdown of KDM5C led to increased autophagy and apoptosis, and reduced xenograft tumor growth in vivo. The observed effects were related mechanistically to the negative modulation of *PFDN5* expression by reducing the level of H3K4me3 in *PFDN5* promoter region. In effect, KDM5C could alleviate the inhibitory effect of PFDN5 on the expression of c-MYC target genes. Indeed, KDM5C knockdown led to the downregulation of Vimentin and N-cadherin expression, both in vitro and in vivo [104]. Moreover, the overexpression of KDM5B in cervical cancer cells reduced apoptosis, and promoted cell migration and invasion by elevating the expression of MMP2/9 [105]. Similarly, KDM5C silencing reduced the growth of gastric xenograft tumors [106].

### 2.6. KDM6A/B (UTX/JMJD3)

KDM6 proteins catalyze the demethylation of H3K27me2/3, which may lead to transcriptional activation [3,8]. Depending on tumor type, KDM6A (UTX) can either promote or suppress tumorigenesis, but it is usually considered as anti-neoplastic [107]. However, the downregulation of KDM6A by shRNA suppressed proliferation and promoted apoptosis in patient-derived glioblastoma cells. Mechanistically, KDM6A decreased H3K27me2/me3, which activated the expression of periostin—a protein implicated in cancer cell metastasis and EMT [108]. Moreover, KDM6A, in coordination with lysine methyltransferase KMT2B, regulated the self-renewal and chemoresistance of NSCLC stem cells by activating the Wnt/β-catenin signaling pathway. Importantly, the inhibition of KDM6A and KMT2B reduced tumor growth and prevented recurrence in xenografted animals [109]. Another study provided evidence that KDM6A is implicated in driving metastasis in TNBC associated with *KMT2C/D* loss by modulating the expression of matrix metalloproteinase MMP3 [110]. These data point to the complexity of epigenetic control of gene expression and the importance of taking into consideration the cross-talk between epigenetic proteins when designing interventions.

In contrast, the inactivation of KDM6A induced colorectal cancer cell survival in vitro and tumor growth in vivo. It was shown that KDM6A knockdown promoted the binding of HIF1α transcription factor to the *LDHA* promoter, forcing glycolytic activity and lactate production [111]. In addition, mucoadhesive mRNA nanoparticles used for the intravesical delivery of *KDM6A*-mRNA in mice bearing orthotopic *Kdm6a*-null bladder cancer reduced bladder size and weight, and significantly decreased lymph node volume and Ki-67 expression [112]. Thus, the role of KDM6A seems highly context-dependent, and its inhibition or activation may be required depending on the tissue of origin and genetic makeup.

On the other hand, KDM6B is considered as oncogenic. The shRNA-mediated KDM6B depletion led to reduced tumor volume and weight in gastric cancer xenograft mice. Importantly, this also led to reduced hepatopulmonary metastasis, which was associated with the reduction in N-cadherin and promotion of E-cadherin expression. In addition, it was observed that the common risk factor of gastric cancer—H. pylori infection, correlated with elevated KDM6B expression in gastric cancer patients and in vitro cell lines [113]. GSK-J4—a potent KDM6B inhibitor—reduced the viability and invasive capacity of lung adenocarcinoma cells, altering the expression of metastasis-related genes: upregulating the expression of suppressors of metastasis (fibrinogen alpha—FGA, Nidogen-2, ITIH2 protease inhibitor) and reducing the expression of pro-metastatic genes (Perioxidesin—PXDN, Fucosyltransferase-1—FUT1) [114]. KDM6B was shown to modulate stemness phenotype in epithelial cancer cells by modulating the expression of *SOX2* and *CD44* [115]. The depletion of KDM6B reduced the stemness of hepatocellular cancer cells, and JIB-04, a KDM4/6 inhibitor, significantly reduced the level of expression of stem cell marker genes (*CD133/PROM1, CD44, LGR5, EpCAM*) [73].

### 2.7. KDM7B (PHF8, JHDM1F)

KDM7B (or more commonly—PHF8) catalyzes the demethylation of H3K9me1/2, H3K27me1/2, and H4K20me1/2 [8]. PHF8 showed overexpression in many cancer types, including lung squamous cell carcinomas and adenocarcinomas, colon adenocarcinomas, and metastatic melanomas [77,116]. Apart from targeting histones, PHF8 may also demethylate non-histone proteins, e.g., YY1. Both the knockdown and the pharmacological inhibition using iPHF8 led to reduced proliferation of lung and colon cancer cells, and reduced xenograft tumor growth in vivo [77]. On the other hand, PHF8 knockdown did not affect the proliferation of melanoma cells in vitro or xenograft tumor growth in vivo, but significantly reduced melanoma cells’ invasive capacity. PHF8 was found to activate the transcription of several genes associated with invasion and metastasis, including genes encoding matrix metalloproteinases or integrins. Importantly, the pro-metastatic activity was related to the upregulation of TGFβ/SMAD signaling by upregulating the expression of TGFβ ligands and receptors due to reduced level of H3K9me and H4K20me in their promoter regions [116].

PHF8 showed increased expression in chronic myeloid leukemia, and it was found to mechanistically promote the expression of the *BCR:ABL* fusion gene by demethylating H3K9me1/2 and H3K27me. The silencing of PHF8 caused cell differentiation and reduced cell proliferation, which was associated with the downregulation of ERK and STAT5 signaling [76]. It was also found that PHF8 was highly expressed in CRC patients showing *KRAS* or *BRAF* mutations, which correlated with poor prognosis. PHF8 promoted the expression of KRAS, BRAF, and c-MYC by reducing the level of H3K9me2 within their promoter regions. Importantly, PHF8 was also associated with metastasis by modulating the expression of factors affecting cell migration and invasion: N-cadherin, Slug, Snail, Vimentin, and MMP2/9. PHF8 knockdown effectively delayed the growth of *KRAS*- or *BRAF*-mutant tumors in vivo [117].

### 2.8. Other KDMs

Less is known about the role of other KDMs in cancer. KDM8 (JMJD5) is believed to target H3K9me1 and H3K36me2, but its demethylase activity is uncertain [118]. KDM8 showed negative correlation with EGFR expression in lung cancer patients. Importantly, KDM8 non-epigenetically promoted EGFR protein (both wild type and mutant) degradation via the ubiquitin/proteasome pathway. Moreover, KDM8 reduced the proliferation and motility of lung cancer cells, and increased sensitivity to gefitinib, which pointed to its tumor suppressive function [119]. Similarly, another study reported that the knockdown of KDM8 promoted lung cancer cell proliferation, and xenograft tumor growth in experimental mice [120]. In addition, the KDM8 reduced cell proliferation and promoted apoptosis in pancreatic cancer cells [121]. On the other hand, dimethyl esters of 5-aminoalkyl-substituted derivatives of 2,4-pyridine dicarboxylic acid (compounds 19i and 19j) inhibited the catalytic activity of KDM8, and reduced the viability of several cancer cell lines [122]. These findings may point to the distinct functions of KDMs depending on their enzymatic versus non-catalytic activity [118]. On the other hand, the knockdown of KDM8 reduced the proliferation of glioblastoma cells [123]. Moreover, it was hypothesized that the anti-proliferative and pro-apoptotic effects of allyl isothiocyanate could be attributed to the downregulation of KDM8 and concurrent elevation in H3K36me2 in oral cancer cells [124]. Interestingly, another study reported that the expression of KDM8 was associated with cancer progression in OSCC patients, and the downregulation of KDM8 was important for the anti-cancer effects of silibinin in a model of patient-derived xenograft tumors [125]. These contradictory observations may point to different roles of KDM8 in various cancers.

KDM9 (RSBN1) is believed to catalyze the demethylation of H4K20me2/3, which leads to transcriptional activation. It was shown to be implicated in gastric cancer progression and metastasis mediated by the overexpression of *Linc01711*. This long non-coding RNA molecule was shown to stimulate the binding of KDM9 to gene promoter regions and regulate gene expression, including the expression of lysophosphatidylcholine acyltransferase 1 which plays a role in cholesterol metabolism [126]. The expression of *KDM9* was associated with pathological grade in meningiomas [127].

JARID2 is a protein showing structural homology with KDM5 demethylases but lacking apparent catalytic activity. It plays important roles in regulating the function of epigenetic complexes, especially the Polycomb repressive complex 2 (PRC2), which promotes H3K27me3 [118]. There are contradictory observations regarding the role of JARID2 in OSCC. Reduced JARID2 expression was observed in two OSCC cell lines when compared with normal keratinocytes [128], while The Cancer Genome Atlas (TCGA) data analysis pointed to elevated expression of JARID2 in OSCC patients [129]. Moreover, the knockdown of JARID2 using siRNA led to increased motility of HSC-3 cells [128], but reduced the migration and invasion of HN30 and SCC25 cells [129]. On the other hand, increased expression of JARID2 was detected in breast cancer patients, and its knockdown led to reduced cell proliferation, motility, and stemness in vitro [130]. Thus, current contradictory evidence does not allow us to consider JARID2 as unequivocally oncogenic, and more studies are necessary to dissect its roles in different cancers.

## 3. The Effect of KDM Inhibition on Cancer Cell Metabolism

Metabolic alterations are well-known to play a hallmark role in carcinogenesis [1]. This is exemplified by the enhanced activity of glycolysis, which supports the increased requirement for nutrients necessary for cell growth and proliferation, and is associated with the elevated production of lactate (the Warburg effect) [131,132]. JmjC KDMs rely on the availability of 2-ketoglurate, which is produced in the Krebs cycle following the oxidation of glucose, fatty acids, and glutamine and other amino acids. Moreover, the necessary presence of oxygen makes KDMs relatively sensitive to hypoxia, which is frequently observed in solid tumors [4]. Thus, metabolic alterations and changes in KDM activity may be interdependent. On the other hand, by interacting with transcription factors and affecting histone methylation, KDMs may modulate the expression of metabolic enzymes.

Indeed, several KDMs, including LSD1, emerged as important regulators of energetic metabolism. A recent study showed that glioblastoma-initiating cells that were characterized by a glycolytic phenotype were sensitive to pharmacological LSD1 inhibition. LSD1i led to the reduction in basal and compensatory glycolysis, and caused mitochondrial distress [43]. The depletion of KDM4C suppressed ATP production and mitochondrial oxidative metabolism in prostate cancer cells, leading to the induction of a more quiescent phenotype. This was associated with the reduced expression of glycolytic (*HK2, PFK1, LDHA*) and TCA cycle (*MDH1, PDHA, ACO1*) genes. These effects were mediated by altering the expression and transcriptional activity of the metabolic master regulator c-MYC [88]. Moreover, the anti-cancer effects of caloric restriction could be potentiated by LSD1 inhibition in PDX models of acute myeloid leukemia and triple-negative breast cancer [133]. Glycolysis can be regulated also by KDM5A, which affects the level of H3K4me3 in the promoter region of fructose-1,6-diphosphatase (*FBP1*) gene. FBP1 inhibited glycolysis in hepatocellular carcinoma cells, and its expression was regulated by FOXP2 which reduced the KDM5A-mediated loss of H3K4me3 from *FBP1* promoter region. This led to the suppression of cell proliferation and invasion [134]. On the other hand, C70—a KDM5 inhibitor—reduced mitochondrial respiration in breast cancer cells, pointing to significant modulation of the expression of mitochondrial metabolism proteins by KDM5. Indeed, KDM5A seems to be the key regulator of c-MYC-driven transcription [101]. In addition, MC3324, a dual inhibitor of LSD1 and KDM6A/B, increased H3K4me2 and H3K27me3, followed by growth arrest and enhanced apoptosis of prostate cancer cells, downregulation of AR, and impairment of cellular metabolism. It reduced basal and maximal respiration, ATP production, and content of different proteins and lipids, e.g., phosphocholine [135]. KDM6B was shown to occupy the HIF1 promoter, suggesting its role in hypoxia response [115]. Cell detachment from the extracellular matrix was shown to cause a switch towards glycolysis dependence, as shown by increased expression of *GLUT1/3*, *HK2*, *PFK*, *PGAM1*, *PDK1*, and *LDHA*. These changes were regulated transcriptionally by KDM6, and KDM6 inhibition using GSK-J4 significantly reduced the expression of these glycolytic genes. GSK-J4 reduced the glycolytic phenotype and induced oxidative stress by affecting glutathione level and superoxide dysmuthase (SOD) activity. Overall, this led to the promotion of apoptosis in detached anoikis-resistant cancer cells [136]. In addition, JARID2 was shown to promote the expression of glycolysis-related genes in breast cancer cells, while its knockdown suppressed glycolytic activity [130].

The silencing of LSD1 was associated with the reduction in the expression of lipid metabolism enzymes (FASN, SREBP1/2) in hepatocellular cancer cells [15]. Moreover, SP2509 LSD1 inhibitor affected fatty acid metabolism and induced lipid droplet accumulation via impairment of lipophagy in pancreatic ductal adenocarcinoma (PDAC) cells. It was observed that cells exposed to 2-deoxyglucose (glycolysis inhibitor) were more sensitive to LSD1 inhibition, because LSD1i disturbed mitochondrial fatty acid oxidation on which cells with suppressed glycolysis depended [137]. In TNBC cells FLAD1 protein was shown to increase the activity of LSD1, which induced the expression of genes related to fatty acid and cholesterol metabolism (*FASN, ACC1, SCD, HMGCR*) by affecting the level of H3K4me2 and H3K9me2 in their promoter regions. LSD1 inhibition reduced the expression of lipogenic genes, including the regulator of cholesterol synthesis—*SREBP1*. Interestingly, TNBC cells showing higher expression of FLAD1 were more sensitive to LSD1 inhibition both in vitro and in vivo [138]. The overexpression of KDM5B in cervical cancer cells and xenograft tumors led to the accumulation of triglycerides and cholesterol in lipid droplets, by decreasing the expression of carnitine palmitoyltransferase IA and increasing the expression of monoglyceride lipase. Moreover, it increased mitochondrial oxidative metabolism [105]. In addition, PHF8 was shown to repress the expression of electron transport chain genes but not TCA or pentose phosphate pathway genes. This required the interaction with YY1 transcription factor. Moreover, PHF8 knockdown led to reduced production of mitochondrial and cytoplasmic reactive oxygen species [77].

## 4. The Inhibition of KDMs and Modulation of Tumor Microenvironment

The tumor microenvironment (TME) forms a niche which supports the survival and growth of cancer cells. It is composed of many types of non-tumor cells, which support cancer cells by secreting growth-stimulatory, proangiogenic, and immune-suppressive factors. Among stromal cells, cancer-associated fibroblasts (CAFs) and tumor-associated macrophages (TAMs) seem to play the most important roles in promoting neoplasia progression [139]. Alterations in KDMs activity may affect the function of most cell types present in TME by modulating cellular plasticity, intracellular processes, or by affecting the intercellular communication.

The inhibition of KDM6B with GSK-J4 prevented the adhesion of mantle cell lymphoma cells to stromal cells, and the effect was mediated by the increase in the level of H3K27me3 in the promoter regions of NF-κB genes and decreased RelA/B expression and nuclear translocation. This was associated with reduced expression of CCR7 chemokine receptor. By downregulating NF-κB pathway, GSK-J4 could reduce cell adhesion, survival, and drug resistance acquisition [69].

KDMs can be engaged in the intercellular cross-talk among cells present in the TME. IL-6 derived from TNBC cells was shown to promote cell transition towards CAFs. Mechanistically, cancer cell-derived IL-6 led to the activation of the STAT3/NF-κB-p50 axis in CAFs, which subsequently upregulated KDM2A expression. In effect, CAFs secreted chemokines (CXCL2, CXCL5, IL-8) which acted on macrophages, stimulating their M2 polarization, which is associated with immune suppression. Indeed, TCGA data showed that tumors characterized by higher stromal KDM2A level were associated with the enrichment in M2 TAMs. Furthermore, M2 TAMs secreted CCL2, which reciprocally acted on TNBC cells, leading to the activation of CCR2 signaling, and the development of paclitaxel resistance [140]. Another study showed that M2 macrophages are characterized by higher expression of KDM2B, which leads to the increased production and secretion of IL-6 by TAMs. This affected the migration and invasion of liver cancer cells, and the overexpression of KDM2B in TAMs led to increased migrastatic potential in vivo [141]. Hypoxia was shown to promote M2 polarization of macrophages, which was associated with increased expression of KDM3A. This led to the production and secretion of VEGF, which activated Akt signaling pathway and enhanced proliferation of ovarian cancer cells [142].

M2 macrophages were shown to secrete CXCL1, which upregulated the activity of KDM6B in cervical cancer cells. The paracrine CXCL1-mediated increase in the activity of KDM6B reduced the level of H3K27me3 in the promoter region of *PFKFB2* gene, leading to its induction. This enhanced the activity of glycolysis and lactate production, and stimulated cell proliferation and migration. Indeed, xenograft tumors containing M2 macrophages showed enhanced growth, which could be reduced by KDM6B knockdown [143]. Interestingly, one study reported that KDM6B protected THP-1 macrophages from the induction of M2 polarization by the co-culture with TNBC cells, while KDM6B knockdown further promoted M2 polarization by activating β-catenin [144]. On the other hand, epithelial cancer cells infected with HPV11 showed the activation of KDM4A. The cells promoted the M1 polarization of macrophages in a paracrine way, and *KDM4A* knockout delayed this process [145].

## 5. The Inhibition of KDMs and Immune Response Modulation

Immune evasion is one of the hallmarks of cancer [1,146]. Cancer cells can avoid eradication by immune cells, e.g., cytotoxic CD8+ T cells, due to immunoediting, but also because of the production of immune suppressive factors, or by attracting and supporting immunosuppressive inflammatory cells, including regulatory T cells [146]. The recently introduced immune checkpoint blockade (ICB) therapies, which can block immune evasion by interrupting the interaction between PD-L1 present in the membrane of cancer cells and PD-1 on T cells, showed limited clinical benefit. However, their efficacy can potentially be augmented by the combined use with other immunomodulatory chemicals, including KDMi [147]. ICB therapy is more effective in patients who present with immune-inflamed (“hot”) tumors, while may lack efficacy in patients presenting with “cold” tumors, which are not infiltrated by cytotoxic immune cells. This TME phenotype can be modulated by various factors, including epigenetic modulators [148].

The immunomodulatory activity of LSD1 is well documented [149,150,151]. Lsd1 inhibition was shown to promote Pd-l1 expression in a mouse model of 4NQO-induced OSCC, and a combination of SP2509, an Lsd1 inhibitor, and anti-Pd-1/anti-Pd-l1 antibodies additively reduced tumor growth in this in vivo model. Moreover, SP2509 promoted tumor infiltration by CD8+ T cells [33]. Similarly, SP2509 showed synergistic anti-cancer effects in a syngeneic model of xenograft breast tumors. LSD1 inhibition was shown to promote the secretion of CXCL9 and CXCL10 chemokines, which could be responsible for the observed enhancement of CD8+ T cell migration [152]. LSD1 is an important player in the regulation of PD-L1 expression. Indeed, a positive correlation between the expression of LSD1 and PD-L1 was found in gastric cancer patients. In addition, the expression of PD-L1 was decreased by novel LSD1 inhibitors—acridine-based DXJ-1, phenothiazine-based 3s, and quinazoline-based compound Z-1—in gastric cancer cells, and in xenograft tumors [153,154,155]. Moreover, LSD1 may significantly affect T cell function. LSD1 inhibition using DXJ-1 or its more potent derivate—5ac—stimulated T cell killing of gastric cancer cells by co-cultured T cells, which showed elevated expression of CCR7, IFNγ, IL-2, and TNFα [153,156]. The level of expression of LSD1 negatively correlated with the infiltration of tumors with CD8+ T cells in gastric cancer [157], or hepatocellular carcinoma [158] patients. In line with this, the knockdown or pharmacological inhibition of LSD1 using compound Z-1 stimulated T-cell killing of gastric cancer cells in vitro, and promoted tumor infiltration by CD4+/CD8+ T cells in tumor xenografts in syngeneic mice [155,157]. In addition, the pharmacological inhibition of LSD1 with SP2509 increased the serum level of several cytokines (IFNβ, IFNγ, IL-9), promoted the infiltration of tongue tumors with activated (CD4+, CD8+, CD45+) T cells, and reduced the percentage of immunosuppressive (CD25+) T cells in experimental mice [34]. Similarly, bomedemstat in combination with anti-PD-1 antibody significantly suppressed the growth of NSCLC xenograft tumors in syngeneic mice, and improved tumor infiltration by CD8+ T cells. The effects were associated with the activation of NOTCH, IL-2/STAT5, IL-6/STAT3, TNFα, IFNα, IFNγ signaling pathways, and upregulation of antigen presenting proteins [159]. In another study, it was shown that the combination of LSD1 inhibitor and anti-PD-1 antibody was especially effective in xenograft NSCLC tumors with lower expression of Trim35—an E3 ligase which causes LSD1 ubiquitination and inactivation [160].

The treatment of T cells with an LSD1 inhibitor OG-L002 stimulated the secretion of antineoplastic IFNγ and Granzyme B, and increased the percentage of activated CD8+ T cells, while reducing the percentage of exhausted T cells. Moreover, treated T cells promoted apoptosis in liver cancer cells. In vivo, OG-L002 suppressed liver cancer xenograft growth in syngeneic mice, and increased tumor infiltration with activated CD8+ T cells. Importantly, LSD1 inhibition synergistically improved the effects of anti-PD-1 therapy in experimental animals [158]. Priming with LSD1 inhibitor GSK2879552 followed by concurrent systemic treatment with LSD1i and anti-PD-1 antibody showed excellent therapeutic response in xenograft tumors in syngeneic mice, leading to prolonged response to PD-1 blockade. It seems that preventing T cells exhaustion and promoting intratumoral T cell expansion may be the key aspects of the in vivo effectiveness of LSD1 inhibitors [161]. Indeed, LSD1 inhibition may be important for the rejuvenation of T cells. The prolonged stimulation of T cell receptor (TCR) could drive T cell exhaustion, which could be further maintained by the interaction between PD-1 receptor present in the membrane of T cells, and PD-L1 present on the surface of cancer cells, which can be targeted by anti-PD-1 therapeutic strategies. The inhibition of LSD1 (GSK2879552, ORY1001) during TCR stimulation could prevent T cell exhaustion, which probably relied on the modulation of IL-2/STAT5 signaling which reduced PD-1 level in T cells [162]. It was shown that the treatment of isolated T cells with LSD1 inhibitors during ex vivo activation and expansion prevented exhaustion and led to increased anti-tumor effects of adoptively transferred T cells in melanoma xenograft bearing mice [162,163].

Chemokine expression is epigenetically regulated. The FBXO24-mediated depletion of LSD1 led to increased expression of CCL5 and CXCL10 in breast cancer cells, which was associated with increased level of H3K4me2 in the promoter regions of their genes [39]. Moreover, it was shown that *NSD1* deficient tumors were characterized by reduced CXCL9 and CXCL10 levels, and lack of infiltrating T cells. NSD1 is a histone methyltransferase catalyzing H3K36me2, and *NSD1* loss-of-function mutations are observed in a group of HNSCC patients. NSD1-mediated H3K36me2 antagonizes H3K27me3 by the PRC2 complex, and the loss of NSD1 led to decreased H3K36me2 and increased H3K27me3 within chemokine gene promoters, causing their downregulation. The knockdown of KDM2A, the demethylase targeting H3K36 methylation, restored H3K36me2 level and increased chemokine expression in NSD1-deficient cells, which was associated with increased T cell infiltration and reduced tumor growth in immunocompetent mice [164]. In a syngeneic lung cancer model, the pharmacological KDM4 inhibition with SD70 increased CXCL10 expression and promoted CD8+ T cell infiltration, causing a transition from “cold” tumors into “hot” tumors, which responded to anti-PD1 immune checkpoint blockade treatment. In fact, the strongest antitumor effects were observed for a triple combination of SD70 with anti-PD1 antibody and radiotherapy [165].

The cGAS-STING pathway plays an important role in stimulating innate immune response. The cyclic GMP-AMP synthase (cGAS) protein acts as a sensor of nucleic acids of viral or bacterial origin, but it also reacts to endogenous retroviral elements (ERV). Upon binding dsDNA, cGAS stimulates the activity of the stimulator of interferon genes (STING) protein, which promotes the nuclear translocation of interferon regulatory factor 3 (IRF3) and its binding to NF-κB. This leads to the induction of the expression of type I interferons (IFN-I) and other inflammatory factors. Epigenetic factors are implicated in the regulation of the expression of ERVs, and the expression of downstream pro-inflammatory genes [166]. LSD1 was shown to regulate dsRNA expression and type I IFN response in leukemic cells [133]. Indeed, the stabilization of LSD1 by CDK9 led to the silencing of the expression of ERVs, and the suppression of IFN response, while LSD1 knockdown stimulated chemokine (CCL5, CXCL9, CXCL10) expression [167]. LSD1 inhibition using GSK-LSD1 increased the expression of human ERV, leading to dsRNA accumulation and promotion of IFNβ signaling. This was associated with enhanced production of chemokines (CCL5, CXCL10, CXCL11, IL-15) and increased CD8+ T cell infiltration, causing Granzyme B-mediated induction of breast cancer cell apoptosis [168]. It was shown that the overexpression of LSD1 in ESCC cells blocked the activity of the cGAS-STING pathway in stromal lymphocytes via suppression of NF-κB-dependent expression of pro-inflammatory genes [169]. The expression of IFN response genes negatively correlated with KDM3A expression in gastric cancer patients, and *KDM3A* knockout elevated the expression of CXCL10, ISG15 or IFNβ. The stimulation of IFN response by *KDM3A* knockout was associated with the increased expression of ERVs, and the reduction in tumor growth by *KDM3A* knockout was dependent on the modulation of type I IFN pathway. Importantly, xenograft tumors from cells with *KDM3A* knockout were characterized with increased CD8+ T cell infiltration, and elevated expression of Granzyme B and IFNγ. Moreover, the effects were further enhanced when *KDM3A* ablation was combined with anti-PD1 therapy [170]. Reduced activity of the cGAS-STING pathway, together with decreased level of CXCL9, CXCL10, CXCL11, IFNβ, and IL-10 were observed in castration-resistant prostate cancer. The knockdown of KDM4A in a patient-derived xenograft model of prostate cancer led to cGAS-STING activation, together with the increased expression of the aforementioned cytokines [87]. KDM4 inhibition using JIB-04 increased the cellular content of dsDNA in the cytoplasm, which stimulated the cGAS-STING pathway and subsequently triggered type I IFN response. In addition, JIB-04 stimulated CD8+ T cell infiltration in xenograft tumors in syngeneic mice. TCGA data analysis pointed to the association between KDM4B expression and immunosuppressive TME, and indeed, anti-PD1 therapy non-responders showed higher expression of KDM4B [171]. Similarly, KDM4A was overexpressed in ESCC patients who did not respond to anti-PD1 therapy, and application of KDM4 inhibitor (KDM4-IN-4) followed by anti-PD1 therapy led to pronounced suppression of tumor growth in vivo [84]. KDM5 inhibitor KDOAM25 also stimulated the release of mitochondrial dsRNA into cytoplasm, and subsequently induced type I IFN signaling in TNBC cells [172]. KDM5B was shown to bind to the promoter region and repress the transcription of STING, which acts as a stimulator of interferon genes. In line with this, a novel CPI-455-based PROTAC degrader of KDM5B activated type I IFN signaling in prostate cancer cells [11]. In addition, KDM5A was shown to negatively correlate with CD8+ T cell infiltration in ovarian cancers, and the knockout of *KDM5A* reduced ovarian tumor burden in immunocompetent mice. This was associated with reduced expression of PD-1 and enhanced infiltration of tumors with activated CD8+ T cells [173]. Furthermore, KDM5B depletion regulated innate immunity by increasing STING expression, which was associated with increased expression of IFNγ, and enhanced T cell infiltration in a pancreatic cancer in vivo model [174].

PHF8 inhibited immune response by reducing the percentage of M1 TAMs, CD4+, and CD8+ T cells, and increasing the number of M2 TAMs in CRC. Moreover, it reduced the expression of interferon regulatory factors (IRF1/7/9), and chemokines (CCL5, CXCL10). It was also shown to be responsible for the transcriptional upregulation of PD-L1 expression, and PHF8 knockdown decreased PD-L1 expression by reducing H3K4me3 and increasing H3K9me2 within its promoter region in CRC cells. Importantly, the pharmacological inhibition of PHF8 with daminozide synergized with anti-PD1 antibody in vivo, leading to increased tumor infiltration with CD4+ and CD8+ T cells, and slower CRC tumor growth [117]. PHF8 was found to regulate the expression of endogenous retrotransposons, and its depletion improved the outcome of anti-PD-1 therapy [175]. In a similar manner, KDM3A was shown to stimulate PD-L1 expression via interaction with c-MYC, and KDM3A inhibition using HG (3-(hydroxypicolinoyl) glycine) led to PD-L1 downregulation. Individually applied HG was not effective in reducing tumor growth in vivo in orthotopic models of TNBC and PDAC. However, nanoparticles containing HG and PPA (polyinosinic:polycytidylic acid)—a toll-like receptor 3 agonist—effectively remodeled TME, promoting the repolarization of immunosuppressive M2 macrophages into immunostimulatory M1 macrophages, enhancing the phagocytic capability of macrophages, activating cytotoxic T cells, and causing a striking reduction in tumor growth and the formation of lung metastases [176].

## 6. The Use of KDMi for Chemo- and Radiotherapy Sensitization

Recent studies provided robust evidence for the importance of KDM inhibitors in the modulation of sensitivity to classical chemotherapeutic agents, molecular targeted therapeutics, and radiotherapy. This is most probably associated with the potential of KDM inhibitors to affect DNA damage response and modulate the activity of signaling pathways responsible for cell cycle regulation and apoptosis. Accordingly, bioinformatic analyses showed that a KDM genes-related signature predicted response to chemotherapy and immunotherapy in gastric cancer patients [106].

The significance of LSD1 in therapy resistance has been especially well-researched. The knockdown or pharmacological inhibition of LSD1 (using SP2509) sensitized TNBC cells to paclitaxel [152]. The response to doxorubicin in gastric cancer cells was augmented when exposure to doxorubicin was preceded by 24-h-incubation with GSK-LSD1 [177]. This implies that molecular alterations caused by priming with epigenetic modulators may significantly affect cellular response to therapeutics. Further in vivo tests using athymic BALB/c nude mice xenografts confirmed that a combination of GSK-LSD1 and doxorubicin treatment led to the highest reduction in tumor volume and weight. Similar results for the combination of GSK-LSD1 and doxorubicin were described in mice bearing thyroid cancer [42] or TNBC xenografts [138]. In another study, the activity of NCD38 and SP2509 LSD1 inhibitors in combination with chemotherapeutics were analyzed in ovarian cancer cell lines. Synergistic reduction in cell viability was detected for the combinations of LSD1 inhibitors with cisplatin or carboplatin [178]. Moreover, synergy in apoptosis induction was measured for NCD38 or SP2509 co-treatment with cisplatin, and confirmed in vivo by striking tumor volume and weight reduction (>90% compared to the vehicle control group). A similar study based on a mouse ES2 cells xenograft of ovarian cancer showed that NCD38, combined with LY500307 (inhibitor of estrogen receptor β), also significantly improved the anti-cancer effects of single compounds [179]. In MYCN-expressing neuroblastomas, the overexpression of LSD1 promotes an undifferentiated cellular state and correlates with poor prognosis. Thus, drugs targeting LSD1 can help to treat resistant neuroblastoma cells. Indeed, a new small molecule inhibitor of LSD1—compound 48 (N-(2-(1H-tetrazol-5-yl) phenyl) benzenesulfonamide derivative), synergistically reduced the viability of neuroblastoma cells exposed to bortezomib, a proteasome 26S inhibitor [180]. JIB-097 is a dual LSD1 and histone deacetylase 6 (HDAC6) inhibitor, which was tested against various cancer types, e.g., in multiple myeloma MM1.S cells-derived mice xenograft, and its combination with bortezomib or pomalidomide enhanced tumor volume reduction [181]. Finally, further advancement in LSD1 inhibition-based therapy can occur due to the creation of novel compounds, e.g., targeting neddylation at lysine 63. This modification promotes the ubiquitination of LSD1 and its proteasomal degradation and, as described for gastric cancer cells, can stimulate sensitivity to chemotherapeutics, like 5-fluorouracil and oxaliplatin [182]. Epigenetic mechanisms affect cell plasticity, which is important for cancer progression and development of resistance. It was shown that CRC with activating *BRAF* mutations, upon treatment with BRAFi and EGFRi, get enriched in enteroendocrine cells, which cause the resistance to targeted therapy. In this scenario, LSD1 inhibition could reduce lineage plasticity and prevent the induction of enteroendocrine cells by a combination of BRAFi and EGFRi, thus leading to sensitization to these drugs [183].

IOX1—the KDM3/4 inhibitor—sensitized cancer cells to micelles containing doxorubicin (OPDOX). It increased the cellular uptake and transcellular absorption of OPDOX. This led to enhanced anti-tumor effects of the combination of OPDOX and IOX1 in experimental mice [184]. KDM4A promoted docetaxel resistance in castration-resistant prostate cancer (C-R PC) cell lines by regulating cytoskeleton remodeling through miR-34a/Stathmin 1/β3-Tubulin axis [185]. KDM4B was overexpressed in this cancer type as well [186]. Therefore, compounds inhibiting KDM4s may be crucial to overcome castration-resistant prostate cancer therapeutic problems. The small molecule KDM4B inhibitor B3 sensitized C-R PC cells and C-R PC xenografts to enzalutamide (nonsteroidal AR inhibitor), and synergistically blocked tumor growth in combination with rapamycin [186]. Plant-derived myricetin was shown to act as a pan-KDM4 inhibitor, and its lactic-co-glycolic acid formulation used with enzalutamide synergistically reduced tumor volume and percentage of Ki-67 positive cells in C-R PC mice xenograft of C4-2B cells [31]. Inhibition of KDM4B can also be used to treat other cancers. For instance, the QC6352 inhibitor strongly reduced the tumor volume of xenografted alveolar rhabdomyosarcoma in mice, and presented synergy when applied with vincristine or irinotecan [187]. The same inhibitor was used for the sensitization of gefitinib-resistant TNBC cells to EGFRi treatment. In this model, KDM4 inhibition with QC6352 restored H3K9me3 around DNA regions responsible for the regulation of *FYN* expression, which was shown to serve as a key mediator of drug tolerance. The combination of QC6352 and gefitinib synergistically reduced xenograft tumor growth in vivo [188]. QC6352 also significantly improved the efficacy of vincristine and irinotecan against neuroblastoma in a patient-derived xenograft model [61]. In turn, KDM4C expression was upregulated in the bortezomib-resistant multiple myeloma KM3/BT2 cells, and could serve as a potential molecular target [189]. Plant-derived molecule luteolin was shown to bind to KDM4C, and in ovarian cancer cells, it significantly decreased proliferation, sphere formation, and expression of markers of cancer stem cells like SOX2, OCT4, and NANOG proteins, and ALDH1 activity. Simultaneously, in the mouse Cavo-3 spheroid cells-derived xenograft, luteolin enhanced paclitaxel and carboplatin’s anti-cancer activity [29]. Furthermore, the knockdown of KDM4C sensitized hepatoma cancer cells to cisplatin [92], and the knockdown of KDM4A effectively sensitized resistant ovarian cancer cells to cisplatin [190]. In addition, KDM4A enhanced temozolomide resistance of glioma cells by modulating ROCK2 kinase and HUWE1 E3 ubiquitin ligase transcription and expression through decreased H3K9me3 and H3K36me3 [191]. The knockdown of LSD1 also sensitized glioma cells to temozolomide, both in vitro and in the patient-derived xenograft model [192].

The overexpression of KDM5B correlated with tumor progression and cisplatin resistance in patients with nasopharyngeal cancer [193]. In detail, KDM5B inhibited the expression of Zinc finger and BTB domain-containing protein 16 (ZBTB16) by decreasing the level of H3K4me3 at its promoter, resulting in increased expression of topoisomerase II-α, and inducing cisplatin resistance. Moreover, it was shown that chemosensitive PDAC cells adapted to the treatment with gemcitabine or oxaliplatin by inducing the expression of KDM5A/C, and indeed, the expression of KDM5A/C was elevated in gemcitabine-resistant PDAC cell lines, while their knockdown sensitized resistant cells to the drug [194]. Similarly, shRNA against KDM5B decreased tumor volume and weight by 60–70% in PDAC mice xenograft, while in combination with gemcitabine, the tumor growth was inhibited much stronger than individually used shKDM5B or gemcitabine [195].

Targeting KDM6A/B also might improve the anti-cancer effects of cisplatin. Indeed, alterations in H3K27 methylation regulate osteosarcoma cell sensitivity to cisplatin, and the combination of GSK-J4—a potent KDM6 inhibitor, and cisplatin reduced viability and activated apoptosis in 143B osteosarcoma cells in vitro, followed by augmented suppression of tumor progression in vivo [196]. Similarly, in refractory testicular germ cells, the co-treatment with GSK-J4 and cisplatin activated p53 tumor suppressor response and synergistically reduced in vivo xenograft tumor growth [197].

The knockdown of PHF8 sensitized chronic myeloid leukemia cells to imatinib [76]. Moreover, KDM7A supported cetuximab resistance in head and neck cancer cells, and its knockdown reduced tumor growth and led to sensitization to cetuximab in vivo [198]. Thus, KDM7A level could constitute a novel biomarker of cetuximab sensitivity.

Overall, many studies point to the necessity of combinatorial therapy to overcome chemoresistance, in order to inhibit multiple signaling proteins that adaptively compensate for each other’s depletion [188]. KDMi can act as excellent chemosensitizers because of the engagement of KDMs in the master regulation of transcriptional programs, which affects several resistance-associated features, as described above.

KDMi can also be applied to increase the radiosensitivity of cancer cells. IOX1 molecule (used for KDM4A inhibition) decreased chromatin accessibility around promoter regions of DNA damage repair genes, and reduced telomerase activity, resulting in the enhancement of radiosensitivity of NSCLC cells in vitro and in vivo. Individually applied radiation therapy (RT) or IOX1 reduced tumor weight by approximately half, but their joint use led to much stronger effects in xenograft mice [199]. KDM4C silencing enhanced RT sensitivity in hepatocellular cancer cells in vitro, which was associated with impairment of homologous repair (HR) pathway [92]. Similarly, *KDM4B* knockout sensitized breast cancer cells to ionizing radiation [200]. Furthermore, KDM4A deletion improved apoptosis induction by radiotherapy in ESCC cells, which was associated with the accumulation of DNA damage marked by γH2A.X foci [84]. In ESCC cells, the inhibition of KDM5B (by shRNA), led to additive increase in radiation-induced apoptosis and G1/G0 cell cycle arrest, and decreased colony formation in vitro. In addition, the combined treatment showed significantly better reduction in tumor growth in xenograft mice, compared to shKDM5B or RT used separately [201]. Furthermore, KDM6 inhibition using GSK-J4 decreased the expression of p53 by increasing the level of H3K27me3 within *TP53* promoter region, and improved radiosensitivity in prostate cancer [202].

RT kills cancer cells by causing double strand breaks (DSB) which exceed the capacity of DNA repair mechanisms. Several KDMs have been implicated in the DNA damage response and promoting DNA repair. In fact, DNA damage requires chromatin structure alterations to enable repair, and KDMs play a role in DNA damage signaling. KDM4B and KDM5B were associated with DSB recognition and homologous recombination (HR) pathway in breast and pancreatic cancer, respectively [98,200]. Moreover, KDM4C silencing reduced IR radiation-induced Rad51-foci formation, pointing to impaired HR repair of DSB in hepatocellular cancer cells [92]. In addition, KDM6A/B were implicated in DNA damage response in acute myeloid leukemia (AML) cells, and the loss of *KDM6A* sensitized cells to poly (ADP-ribose) polymerase (PARP) inhibition using olaparib, pointing to a possibility of synthetic lethality between *KDM6A* loss-of-function mutations—which are observed in AML patients—and pharmacological inhibition of PARP [203]. While olaparib in combination with KDMi did not exert pronounced effects in HNSCC cells, a triple combination of KDM4 or KDM6 inhibitors with olaparib and cisplatin led to a significant accumulation of DSB and induction of apoptosis [204]. Indeed, increased sensitivity to olaparib was observed in HNSCC cases with concurrent reduction in H3K36 methylation (e.g., due to *NSD1* histone methyltransferase mutation) and increase in H3K27 trimethylation. This epigenetic profile was associated with the enhanced formation of DSB and the reduced activity of DSB-repair pathways. In cells characterized by reduced H3K36 methylation, the sensitization to PARP inhibitors (PARPi) could be pharmacologically induced using the KDM6 inhibitor GSK-J4 [205]. NSCLC cells resistant to cisplatin or paclitaxel showed elevated expression of several KDMs (3A, 4B, 5A, 6A), and the knockdown of KDM4B (but not 4A or 4D) or KDM6A most significantly sensitized cells to cisplatin, similarly to pharmacological inhibition using ML324 or JIB-04. Xenograft tumors developed from cisplatin-resistant cells were sensitive to the combination of cisplatin and JIB-04. Mechanistically, the effects were related to changes in the level of expression of DSB repair-related proteins, especially those important in the HR pathway. For instance, JIB-04 blocked HR repair, and led to the accumulation of cisplatin-induced DNA damage [206]. HR deficiency may be caused by *BRCA1/2* mutations in breast and ovarian cancers, which is the basis for the use of PARPi to induce synthetic lethality. Methylstat, a KDM3 inhibitor, pharmacologically induced the HR-deficient phenotype in ovarian cancer cells. In this regard, it synergized with olaparib and sensitized olaparib-resistant cells, leading to enhanced DSB accumulation and cell death, irrespective of *BRCA1/2* mutational background. Other KDM inhibitors (GSK-LSD1, IOX-1—a broad spectrum inhibitor, PFI-90 targeting KDM3B, GSK-J1 targeting KDM6B) showed weaker effects. Importantly, methylstat also mitigated the selection of clones of cells resistant to olaparib, preventing the development of PARPi resistance [207]. In a similar manner, HR-proficient ovarian cancer cases were associated with increased expression of LSD1, which correlated with the level of expression of HR pathway genes. Indeed, LSD1 inhibition using ZY0511 or SP2577 reduced the expression of BRCA1/2 and RAD51, leading to attenuation of HR pathway and induction of DNA damage. LSD1 inhibitors led to synergistic anti-cancer effects when combined with PARP inhibitors (olaparib, niraparib, rucaparib), leading to significant inhibition of the growth of tumors developed from HR-proficient (but not from HR-deficient) cells in experimental animals [208]. Glioblastoma patients who were not responsive to RT were characterized by higher expression of LSD1. In glioma cells, LSD1 was enriched at promoters of DSB repair genes, and LSD1 knockdown attenuated the activity of DSB repair pathways [192].

## 7. The Use of Combinations of KDMi with Other Targeted Therapeutics

Monotherapy is rarely effective in cancer treatment, and thus the search of optimally active combinations of molecules is still an important strategy in oncology. Several KDM inhibitors were tested in combinations with other molecular targeted drugs [6]. Table 3 presents a summary of recent reports where a KDM inhibitor was tested in combination with another drug (potentiator) in order to improve the expected biological effects.

In addition to the combinations of KDMi and other targeted therapeutics, dual-target inhibitors have also been developed and tested [6]. The first group of dual-target inhibitors consists of molecules linking anti-LSD1 activity with the inhibition of another epigenetic modifier. A dual LSD1-HDAC6 inhibitor decreased the proliferation of HCT-116 CRC cells and effectively inhibited the growth of CRC patient-derived organoids [225]. In another study, a derivative of GSK2879552 acting as a dual LSD1-HDAC6 inhibitor was developed, and the compound showed stronger effects on AML cell viability than the parent LSD1 inhibitor. Moreover, the priming of AML cells with the chemical sensitized cells to doxorubicin, leading to significant enhancement in apoptosis induction [226]. Additionally, tumor volume and weight reduction during treatment of castration-resistant prostate cancer with enzalutamide were described in combination with EZH2/LSD1 dual inhibitor ML234 [227]. N-(4-(hydroxycarbamoyl) benzyl)-2-(4-methoxyphenyl) isonicotinamide, named as compound 5e, is a potent LSD1 and histone deacetylases (HDACs) inhibitor [228]. The compound significantly affected viability in leukemia cell lines (MOLT-4 and MV4-11), gastric cancer MGC-803 cells, lung cancer A-549 cells, and colorectal cancer HCT-116 cells already at low concentrations. Further analyses using MGC-803 and HCT-116 cells revealed the induction of apoptosis, reduction in cell migration and invasion, and G2/M cell cycle arrest. Reduced tumor growth in mice xenograft models was also observed. In another study, novel 5-cyano-3-phenylindole-based LSD1 and HDAC dual-inhibitors were discovered, among which 7-(3-(3-Amino-2-methylphenyl)-5-cyano-1H-indol-1-yl)-N-hydroxyheptanamide (compound 20c) exhibited the best anti-cancer activity [229]. Its anti-proliferative activity was assessed in 16 different cell lines revealing IC50 concentrations ranging between 0.69 µM (HCT-166 cells) and 8.03 µM (MDA-MB-231 cells). Suppressed xenograft tumor growth of PAX3-FOXO1 fusion-positive rhabdomyosarcoma and delayed tumor progression were the effects of a dual LSD1 and KDM3B inhibitor, called PFI-90 [55]. One more example of a dual-target epigenetic inhibitor is the MC3324 molecule, which acts against LSD1 and KDM6A. In prostate cancer cells, MC3324 reduced metabolic activity (e.g., basal and maximal respiration, ATP production, and metabolism-related gene expression), followed by growth inhibition and apoptosis induction [135]. WS-384 is a first-in-class inhibitor of LSD1 and DCN1-UBC12 protein–protein interaction, crucial in neddylation and subsequent ubiquitin-dependent protein proteasomal degradation, tested in NSCLC cells. Typical positive anti-cancer effects, like reduced viability and colony formation or increased DNA damage and apoptosis, were shown after exposure to WS-384 [230].

The second group of dual-target inhibitors consists of molecules linking anti-LSD1 activity with the inhibition of a non-epigenetic target. N-ethyl-2,4-dihydroxy-5-isopropyl-N-(3-((methyl (prop-2-yn-1-yl) amino) methyl) phenyl) benzamide (compound 6) is a dual LSD1 and heat shock protein 90 (HSP90) inhibitor, effectively affecting the survival of prostate cancer cells and patient-derived colorectal cancer organoids [231]. Its safety was also tested in the zebrafish in vivo model. Another compound called L-1 is a dual LSD1 and EGFR inhibitor. It reduced tumor size and weight by approximately 75% in the lung cancer H1975 cells xenograft mouse model [232]. The development of resistance to EGFR inhibitors is a significant clinical problem which requires addressing. A recent study characterized a dual inhibitor of EGFR and LSD1 as effective towards EGFRi-resistant NSCLC cells. ZJY-54 irreversibly inhibited mutant EGFR (L858R/T790M), leading to its reduced phosphorylation, and reversibly blocked LSD1, causing the accumulation of H3K4me2 and H3K9me2. The compound suppressed tumor growth in a xenograft model of lung cancer [233].

A novel inhibitor I-25 (MY-943), 2-((3-hydroxy-4-methoxybenzyl) (3,4,5-trimethoxyphenyl) amino)-2-oxoethyl 4-(4-aminophenyl) piperazine-1-carbodithioate, targets both LSD1 and tubulin polymerization. The compound reduced cell viability, migration, and expression of anti-apoptotic and cell cycle-promoting proteins in gastric cancer MGC-803 cell line. Furthermore, xenograft tumor volume reduction was observed without hepatic and renal toxicity induction [234]. Similarly, a novel dual tubulin polymerization and LSD1 inhibitor L-6 (1,2,3-triazole arylamide derivative bearing dithiocarbamate moiety) effectively suppressed colony formation, induced G2/M phase cell cycle arrest, and activated apoptosis in gastric cancer cells [235].

## 8. Perspectives and Conclusions

**Figure 3:**
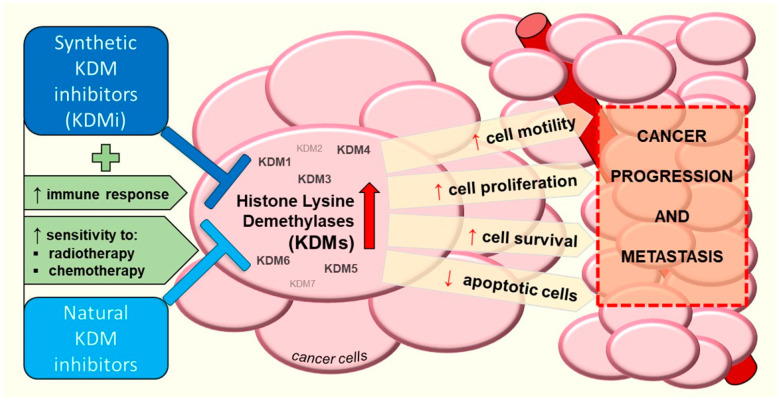
The pro-tumorigenic effects of KDMs justify the therapeutic use of KDM inhibitors.

Most of the currently available evidence points to the significant activity of KDM inhibitors towards key cancer phenotypes, including abnormal proliferation, increased motility associated with the danger of invasion and tumor spread, neovascularization, shaping and adaptation to the altered microenvironment, therapy resistance, and immune control and evasion (Figure 3). However, it must be remembered that some reports showed that the inhibition of KDMs may not be beneficial in cancer control, making it a misguided therapeutic strategy. For example, KDM2B depletion promoted VEGF-dependent vasculature formation by human umbilical vein endothelial cells [236]. In addition, KDM2A expression negatively correlated with breast cancer cell migration and invasion via downregulation of the EGFR signaling pathway and related inhibition of the downstream ERK/MAPK pathway [237]. On the other hand, LSD1 inhibited the migration and invasion of breast cancer cells via exosomes, and the inhibition of LSD1 promoted the epithelial-to-mesenchymal transition in OM-1 oral cancer cells [238,239]. In a similar manner, KDM5D depletion was associated with increased invasion and metastasis in a prostate cancer model [240]. Future studies should focus on the identification of clear contra-indications for KDMi use. Alternatively, the necessity to combine KDMi with other agents to attenuate unfavorable effects may be considered. Interestingly, the stabilization of KDM5C by inhibiting its neddylation with the use of a small inhibitor—MLN4924, sensitized AML cells to the anti-leukemic activity of lenalidome, a Cereblon protein inhibitor [241]. Thus, while valid in most cases, the inhibition of KDMs is not always the desirable therapeutic strategy.

It is important to better characterize the indications for the clinical application of KDMi. Several studies focused on the analysis of molecular characteristics which point to sensitivity to KDMi. Gastric cancer patients are frequently characterized by frameshift mutations of *TP53* that lead to loss of the nuclear localization sequence (NLS). Lack of functional p53 abrogated the transcriptional repression of LSD1 mediated by p53, and caused LSD1 upregulation, with a subsequent induction of the expression of cyclin A2, and abnormal cell proliferation. Gastric cancer cells with *TP53* frameshift NLS mutations were sensitive to LSD1 inhibitors. Indeed, GSK690 significantly reduced the viability and proliferation of mutant, but not wild-type, cells, and also potently reduced the growth of xenograft tumors in experimental animals. These effects were associated with the elevation in H3K9me2 within *CCNA2* promoter and its reduced expression [242]. Higher LSD1 expression was also observed in BRCA1 depleted tumors, and tumor growth suppression could be exerted by LSD1 inactivation [152]. On the other hand, HNSCC patients showing loss of *NSD1* could benefit from KDM6 or KDM2A inhibition [164,205]. Androgen receptor-negative prostate cancers are difficult to treat, but it has been shown that these cancers are vulnerable to KDM3C inhibition, which can significantly suppress the growth of AR-depleted prostate cancer cells. This was associated with upregulated TNFα signaling [243]. Regarding myeloproliferative neoplasms, it was found that KDM4C knockdown effectively reduced cell proliferation and stimulated apoptosis only in cells bearing JAK2^V617F^ mutation, and not in wild-type cells [244]. Moreover, dexamethasone-resistant acute lymphoblastic leukemia cells, which showed elevated expression of CREBBP protein, were characterized by sensitivity to GSK-J4. Indeed, apoptosis could be induced in ALL cells isolated from relapsed patients by treatment with GSK-J4, suggesting that KDM6 inhibition is a relevant therapeutic option in dexamethasone-resistant relapse ALL patients [222]. Moreover, QC6352 showed higher cytotoxic and cytostatic activity towards MYCN-amplified neuroblastoma cells, indicating that MYCN overexpression can sensitize cells to KDM4i [61]. It was shown that GSK-J4 was more potent in cells exposed to TGFβ induction, indicating that activated TGFβ signaling may be a factor mediating susceptibility to KDM6B inhibition [70]. On the other hand, CRC patients showing *KRAS* or *BRAF* mutations could benefit from PHF8 inhibition [117]. Further research is necessary to clearly identify groups of patients amenable to effective KDMi treatment. Another issue is related to the selectivity of KDM inhibition. It remains to be determined whether selective inhibition of individual KDMs is more advantageous than non-selective inhibition of several KDMs. Potentially, non-selective KDM inhibition (e.g., using JIB-04—a potent inhibitor of KDM4–6) could lead to more pronounced destabilization in gene expression profiles in cancer cells, and thus prevent adaptive responses due to partial functional redundancy between some KDMs.

It is also important to study the mechanisms of resistance to available KDMi. The development of drug resistance in cancer patients is inevitable, and it can sometimes be circumvented by the use of combinatorial strategies. For example, glioblastoma tumor-initiating cells which were resistant to LSD1i could be sensitized to the inhibitor by concurrent knockdown of *PGAP1*. In addition, synthetic lethality with LSD1 inhibition was also observed in the case of the knockdown of genes associated with glutamine (*GLS, GLS2*) or lipid (*MTMR14, INPP4A*) metabolism, or nucleotide synthesis (e.g., RRM2B) [43]. A recent study showed that KDMi may adaptively upregulate the cellular level of KDM proteins. The joint application of small molecular KDM4 inhibitors together with CRISPR-mediated KDM4 depletion not only led to the synergistic enhancement of their individual biological effects in colorectal cancer cells, but also prevented adaptive changes in expression, possibly attenuating the acquisition of a resistant phenotype [245].

**Figure 4:**
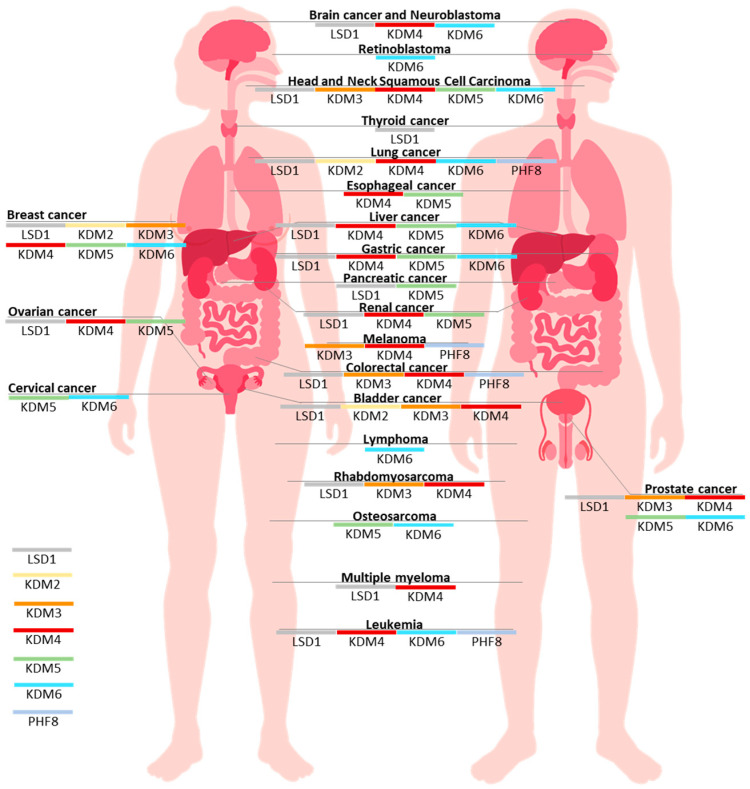
The involvement of KDMs in the development of various cancers. KDMs, for which evidence of functional significance in the relevant cancer type was reported, are listed in each case. Each KDM is marked with a different color (legend on bottom left).

Overall, current evidence strongly supports the identification of KDMs as important pharmacological targets in different types of cancers (Figure 4). Hopefully, the full therapeutic potential of KDMi will be tested in clinical trials in the nearest future, to finally verify the clinical utility of this pharmacological strategy.

## Figures and Tables

**Table 1:** Natural inhibitors of histone lysine demethylases.

Name of Compound	Target KDM	Biological Effects	Reference
Apigenin	LSD1	Apigenin reduced LSD1 expression; reduced hepatocellular cancer cell viability, proliferation, migration, and invasion	[15]
Arborinine	LSD1	Reduced LSD1 expression and increased H3K4me1 in ovarian cancer cell line; reduced cell viability, proliferation, migration, and invasion; decreased tumor volume and weight in mouse xenografts	[16]
		Reduced LSD1 activity with concomitant increased H3K4me1/2 and H3K9me1/2 in clear-cell renal cell carcinoma cell lines; decreased cell viability, colony formation, invasion, and migration; induced apoptosis and S-phase cell cycle arrest	[17]
		Reduced LSD1 activity and increased K3K4me1 and H3K9me1/2; decreased gastric cancer cell viability, also in adriamycin-resistant and vincristin-resistant cells; reduced tumor weight in a mouse xenograft model	[18]
Demethylzeylasteral (T-96)	LSD1	Reduced LSD1 protein level, and elevated H3K4me2 and H3K9me2 levels; reduced cell viability, and diminished tumor growth in a xenograft model of triple-negative breast cancer	[19]
Higenamine	LSD1	Decreased LSD1 activity followed by enhanced H3K4me1/2; activation of apoptosis and induction of cell differentiation in acute myeloid leukemia cell lines; weak inhibition of cell proliferation—higenamine could be used as a pharmacophore for the development of more selective and potent LSD1 inhibitors	[20]
		A higenamine-derived LSD1 inhibitor, FY-56, presents improved effects in vitro; in the mouse acute myeloid leukemia model, FY-56 decreased the number of leukemia cells in peripheral blood and spleen, followed by an increased survival rate of leukemic mice	[21]
Isoforsythiaside	LSD1	Isoforsythiaside is an LSD1 covalent inhibitor; increased the level of H3K4me1/2; reduced breast cancer cells metastasis to lung (mouse MDA-MB-231 cells xenograft)	[22]
Kawain	LSD1	Prevented urothelial carcinogenesis induced by OH-BBN *; increased H3K4me1/2 in cancer cell lines	[23]
Sanguinarine	LSD1	LSD1 reversible inhibition by competition with the FAD site; increased H3K4me2 and H3K9me2 levels; decreased lung cancer cell viability, clonogenicity, and migration; induced apoptosis	[24]
Viscosalactone B	LSD1	Inhibition of LSD1 with increased H3K4me1 and H3K9me1/2; reduced prostate cancer cell viability; decreased tumor volume and weight in a mouse xenograft model without causing toxicity towards major organs, e.g., heart, liver, and kidney	[25]
Caffeic acid	KDM4C	Reduced expression of KDM4C and clonal sphere-forming ability in lung squamous cell carcinoma (LSCC) cells; effects improved by co-inhibition of SOX (shRNA against SOX)	[26]
Genkwanin	KDM4C	Triple-negative breast cancer cells: reduced cell migration, invasion, and self-renewal, decreased expression of SOX2, MMP9, and MMP2; enhanced sensitivity to paclitaxel; reduced cholesterol level	[27]
Kaempferol	KDM4C	Decreased expression of KDM4C and β-catenin in colorectal cancer (CRC) cell lines; reduced CRC cell proliferation and migration; in mouse SW620 CRC cells xenograft kaempferol suppressed lung metastasis and inhibited Wnt/β-catenin signaling pathway	[28]
Luteolin	KDM4C	Luteolin affected the stemness of ovarian cancer stem cells by binding to KDM4C and suppressing KDM4C-mediated H3K9 demethylation in *PPP2CA* promoter region and subsequent inhibition of Hippo/YAP signaling pathway	[29]
Tanshinone I	KDM4D	In gastric cancer cell lines, tanshinone I activated ferroptosis by the inhibition of KDM4D and upregulation of p53 expression	[30]
Myricetin	KDM4	Myricetin is a pan-KDM4 inhibitor; cytotoxicity towards androgen-dependent and androgen-independent prostate cancer cells; enhanced anti-tumor effects in combination with enzalutamide (androgen receptor inhibitor) in vivo	[31]
		Myricetin and its analog increased H3K9me3 in head and neck squamous cell carcinoma (HNSCC) cells, and downregulated ferrochelatase expression by modulating KDM4C, leading to reduced cancer cells dissemination in vivo	[32]

* OH-BBN—N-Butyl-N-(4-hydroxybutyl) nitrosamine.

**Table 3:** The effects of the combinatorial use of KDMi.

Name of KDMi	Potentiator	Biological Effects	Reference
SP2509 (LSD1 inh.)	Verteporfin (photosensitizer)	Enhanced reduction in the growth of tongue tumors induced by 4NQO in mice	[33]
	Anti-PD-1 antibody		
	Ruxolitinib (JAK/STAT inh.)	In vivo sensitization of early T cell progenitor acute lymphoblastic leukemia (ETP-ALL) with induction of apoptosis	[209]
	ABT-199 (BCL2 inh.)		
	2-Deoxyglucose (glycolysis inh.)	Significant reduction in pancreatic ductal adenocarcinoma tumor growth (mice xenograft) by impairment of glucose and lipid energy metabolism	[137]
SP-2577 (seclidemstat— LSD1 inh.)	Encorafenib (BRAF inh.)	SP-2577 sensitized colorectal cancer cells to encorafenib, or encorafenib + EGFRi treatment; SP-2577 enhanced the reduction in tumor growth caused by BRAFi + EGFRi in an orthotopic model of CRC	[183]
GSK-LSD1 (LSD1 inh.)	Sacituzumab govitecan (anti-TROP-2 antibody plus SN-38)	Enhancement of the effects in triple-negative breast cancer cells	[138]
T-3775440 (LSD1 inh.)	Ruxolitinib (JAK/STAT inh.)	Synergistic reduction in viability of Down syndrome-associated myeloid leukemia patient-derived cells	[210]
ORY-1001 (LSD1 inh.)	1S,3R-RSL3 (glutathione peroxidase 4 inh.)	Viability reduction and ferroptosis induction in A549 and H1975 non-small cell lung cancer cells in vitro; significant reduction in mouse xenograft tumor growth compared to single inhibitors	[211]
shRNA against LSD1	siRNA against cMYC	Activation of ferroptosis in lung cancer H1299 and A549 cell lines	[212]
GSK2879552 (LSD1 inh.)	Quizartinib (FLT3 inh.)	In vitro reduction in FLT3-ITD^+^ acute myeloid leukemia cells viability with reduced MYC expression and activity	[213]
	i-BET762 (BET proteins inh.)	In vivo reduction in tumor volume of mouse castration-resistant prostate cancer cells xenograft; decreased MYC signaling	[214]
ZY0511 (LSD1 inh.)	DTP3 (small molecular peptide binding to MAPK kinase 7)	Synergistic viability reduction and apoptosis induction in HepG2 and Hep3B hepatocellular carcinoma cells; the best tumor volume reduction (mice xenograft) for co-treatment	[215]
IOX1 (KDM3A/4C/2A inh.)	ATRA (retinoid agonist)	Synergistic effects on apoptosis induction and decreased cell invasion in bladder cancer cells; reduced metastasis in vivo	[57]
	Bevacizumab (anti-VEGF ab)	Enhanced reduction in tumor growth in colorectal cancer cell xenografts, enhanced infiltration with activated CD4+ and CD8+ T cells	[83]
SD70 (KDM4C inh.)	MI-503 (menin-MLL inh.)	The combination synergistically reduced viability in AML cells with MLL-AF9 rearrangements, induced cell differentiation, reduced the expression of MYC-target genes; the combination effectively reduced leukemia burden in experimental mice	[216]
QC6352 (KDM4C inh.)	Senolytic agent SSK1	The best reduction in tumor volume and weight in the mouse NCI-N87 gastric cancer cells xenograft with the *TP53* gene mutation	[217]
shRNA against KDM5C	shRNA against Yin Yang 1 (YY1) KDM5C-interacting protein	Synergistic reduction in tumor weight in mouse ACHN renal cancer cells xenograft	[218]
PBIT (KDM5 inh.)	15d-PGJ_2_ (PPARγ agonist)	Synergy in C4-2B and PC-3 castration-resistant prostate cancer cells viability reduction	[219]
	SKP2 (S-phase kinase associated protein 2 inh.)	Potentiated reduction in proliferation, migration, AKT signaling pathway activity, and induction of senescence and apoptosis of C4-2B and PC-3 castration-resistant prostate cancer cells	[220]
GSK-J4 (KDM6A/B inh.)	Hesperetin	Reduction in prostate cancer cell viability; inhibition of TGFβ-induced cell migration and invasion	[70]
	SL-176 (WIP1 inh.)	The combination showed synergistic cytotoxicity towards neuroblastoma cells in vitro, and caused enhancement of apoptosis, especially in p53-wild type cells; the combination reduced neuroblastoma growth in zebrafish xenografts	[221]
	Venetoclax Navitoclax (Bcl-2 inh.)	The combination of GSK-J4 with venetoclax or navitoclax showed synergistic anti-cancer effects in acute lymphoblastic leukemia	[222]
	Gilteritinib (FLT3 inh.)	Synergistic reduction in FLT3-ITD^+^ acute myeloid leukemia cells viability; in vivo (mice xenograft), better tumor volume and weight reduction, and apoptosis induction	[223]
Daminozide (PHF8 inh.)	Anti-PD-1 antibody	Synergistic reduction in CRC tumor growth; increased infiltration with activated T cells	[117]
JIB-04 (pan-KDM inh.)	Samotolisib (mTOR signaling inh.)	Sensitization of small cell lung cancer cells H2171 and H524 to mTOR signaling inhibitors	[224]
	Torkinib (mTOR signaling inh.)		

## Abbreviations

The following abbreviations are used in this manuscript:

AML	Acute myeloid leukemia
AR	Androgen receptor
CAFs	Cancer-associated fibroblasts
CRC	Colorectal cancer
EMT	Epithelial-to-mesenchymal transition
ESCC	Esophageal squamous cell carcinoma
HNSCC	Head and neck squamous cell carcinoma
KDM	Histone lysine demethylase
KDMi	Histone lysine demethylase inhibitor
LSD1	Lysine-specific histone demethylase 1A
NSCLC	Non-small cell lung cancer
OSCC	Oral squamous cell carcinoma
PDAC	Pancreatic ductal adenocarcinoma
RCCC	Renal clear-cell carcinoma
TAMs	Tumor-associated macrophages
TME	Tumor microenvironment
TNBC	Triple-negative breast cancer
